# Open-source sub-nanometer stabilization system for super-resolution fluorescence microscopy

**DOI:** 10.1038/s41377-025-02022-6

**Published:** 2025-11-20

**Authors:** Florencia Edorna, Florencia D. Choque, Giovanni Ferrari, Luciano A. Masullo, Piotr Zdańkowski, Guillermo P. Acuna, Philip Tinnefeld, Alan M. Szalai, Lucía F. Lopez, Andrés Zelcer, Fernando D. Stefani

**Affiliations:** 1 Centro de Investigaciones en Bionanociencias (CIBION), Consejo Nacional de Investigaciones Científicas y Técnicas (CONICET), Ciudad Autónoma de Buenos Aires, Argentina; 2 Departamento de Física, Facultad de Ciencias Exactas y Naturales, Universidad de Buenos Aires, Ciudad Autónoma de Buenos Aires, Argentina; 3 Department of Chemistry and Center for NanoScience, Ludwig-Maximilians-Universität München, Munich, Germany; 4 Warsaw University of Technology, Institute of Micromechanics and Photonics, Warsaw, Poland; 5 Department of Physics, University of Fribourg, Fribourg, Switzerland; 6 Swiss National Center for Competence in Research (NCCR) Bio-inspired Materials, University of Fribourg, Fribourg, Switzerland; 7 Present Address: Max Planck Institute of Biochemistry, Planegg, Germany

**Keywords:** Super-resolution microscopy, Imaging and sensing

## Abstract

Recent advances in fluorescence nanoscopy have pushed resolution to the 1–10 nm range, enabling the direct visualization of individual molecules even in crowded biological environments. Achieving this level of precision requires rigorous sample drift control. Techniques such as MINFLUX and RASTMIN, which rely on keeping the sample fixed within an excitation pattern, demand active drift correction to achieve their theoretical nanometer-scale resolution limits. Here, we present an active stabilization system for super-resolution microscopy that delivers sub-nm precision for hours. Featuring a simple optical design, the system can be added as a separate module to any fluorescence microscope. We also provide an open-source control software including a user-friendly graphical interface readily adaptable to different setups. We demonstrate the adaptability and performance of the stabilization system with p-MINFLUX and RASTMIN measurements performed in two different setups, reaching the theoretical Cramér-Rao Bound and resolving ~10 nm distances within DNA origami structures.

## Introduction

Fluorescence nanoscopy has revolutionized life sciences by enabling the study of biological samples at resolutions far beyond the diffraction limit. While most super-resolution fluorescence microscopy techniques routinely achieve a resolution of a few tens of nanometers^1,2^, recent advancements such as MINFLUX^3–5^, RASTMIN^6^, MINSTED^7^ or RESI^8^ have pushed this limit to (or even below) the nanometer regime, entering the realm of structural biology^9–11^. However, reaching such high spatial resolution relies on precise control over multiple factors^12^. Particularly, thermo-mechanical instabilities can introduce a relative displacement between the sample and the microscope objective, known as sample drift, which can ultimately hinder spatial resolution.

Sample drift trajectories can be obtained by monitoring the positions of immobile fluorescent markers or scatterers (fiducials) within the sample or on the coverslip^13–20^, or by leveraging the imaging data itself (e.g., cross-correlation of images or localization events)^21–25^. The obtained drift trajectories can be used to correct drift offline in post-processing or actively during the measurements. The former is simpler as it does not require actuation over the sample position, but it has the disadvantage that sample drift may affect the measurement irreparably, for instance if it moves out of focus. By contrast, active drift correction keeps the sample position stable during the measurement, which is convenient for all super-resolution methods and key for ultra-precise single-molecule localization techniques^26^ that require seconds- to minutes-long measurements over sub-diffraction regions like MINFLUX^3–5^, MINSTED^7^ or RASTMIN^6^.

The strategies used for active stabilization usually differ for the lateral plane and for the axial direction. An efficient route to achieve active focus stabilization consists of using a focused auxiliary beam (at a different wavelength than the one used for fluorescence imaging) directed to the sample under total internal reflection (TIR) illumination^6,15^. When the focus drifts, the image of the reflected beam on a camera is laterally displaced, and this movement can be calibrated and used to maintain the sample in focus. On the other hand, fiducial markers are usually required to stabilize the sample position laterally, and a routine that fits their position with (sub-)nanometer precision is needed to track their displacement over time and reposition the stage^3,5,6^. Fiducial markers can also be used to track and correct axial drift by adding a cylindrical lens in the detection path^19^, generating an astigmatic point spread function. While correcting lateral and axial drift from a single image of the fiducials is in principle simpler, achieving sub-nm corrections in the axial direction requires using strong astigmatism, which in turn leads to errors in the determination of the lateral position of the fiducial markers. Recently, it was shown that achieving sub-nm stabilization in the three directions with this approach requires splitting the signal into two different cameras, one for axial drift correction using astigmatic detection, and the other without astigmatism for lateral stabilization^27^. If the fluorescence experiment involves single fluorophores emitting photons continuously throughout the entire acquisition, astigmatism on this channel can be used to stabilize focus^28^, eliminating the need for fiducial markers. However, in typical nanoscopy experiments, single molecules exhibit photobleaching and blinking, making this approach impractical.

In this work, we present an active stabilization system that can be integrated as an additional module into a fluorescence microscope. Our system allows for 3D stabilization as well as for only focus or lateral drift compensation independently. It is based on an open-source software, which includes a graphical user interface (GUI). The software is hardware-agnostic, meaning it is not tied to any specific hardware components, such as cameras or piezo stages. Instead, it is designed to be highly adaptable to a variety of experimental setups, requiring only access to generic camera and stage control interfaces. We demonstrate that the system can stabilize the sample in position in the three dimensions with sub-nanometer precision for hours, using basic optical components and an affordable piezo actuator. Finally, we demonstrate <1 nm stabilization in RASTMIN and p-MINFLUX measurements in two different setups. Under these optimized conditions, the theoretical limit given by the Cramér-Rao Bound is reached, enabling the reconstruction of DNA origami structures, where distances of 10 nm are fully resolved.

## Results

### General concept

To track and correct the sample drift we use separate strategies for the lateral (XY) and axial (Z) directions. In both cases we use a near-infrared (NIR) laser for illumination as this region of the spectrum is typically available in a fluorescence nanoscopy experiment. To track the lateral displacement, we used 200 nm gold nanoparticles (AuNPs) because they have been reported as effective fiducial markers^29^. Nonetheless, we note that the stabilization system can be used with any other fiducial markers provided they offer stable and detectable signals in the NIR, such as colloidal gold nanorods^4,30,31^ or fiducial markers produced by lithography embedded in the glass substrate^14,16^. Naturally, fiducial markers emitting or scattering in other spectral regions would require a suitable adaptation of the optics. By fitting a 2D Gaussian function to the intensity signal of the light scattered by the fiducial markers, it is possible to estimate the X and Y displacement. To track the axial drift, we used the reflection at the sample interface of a focused beam that is incident in total internal reflection (TIR) and calculated the center of mass of this signal. The lateral and axial (focus) positions were locked using respective control feedback loops.

### Optical setup

**Fig. 1:**
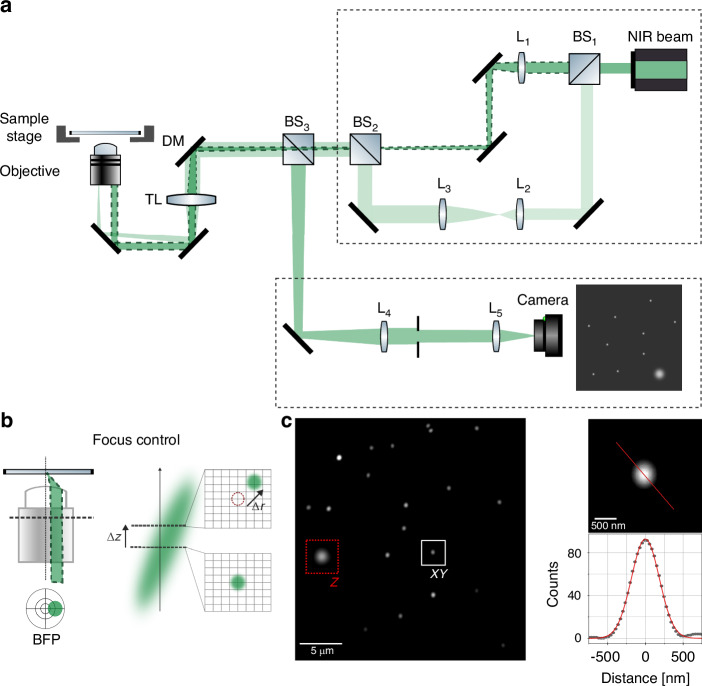
Optical Setup. **a** Scheme of the optical setup implemented for the stabilization system. A beam splitter (BS_1_) splits the NIR light into two paths. In the Z focus path (beam with a dotted border) a lens (L_1_) is matched with the tube lens (TL) of the microscope in a *4* *f* system. For the XY stabilization a telescope formed by L_2_ and L_3_ expands the beam which is focused on the back focal plane (BFP) of the objective by the TL. Both excitation paths are combined by BS_2_. BS_3_ is used to separate excitation light from the signal that will be detected on the camera. In the detection, the system composed by the objective and the TL forms an intermediate image between BS_3_ and L_4_. The *4* *f* system formed by the TL and L_4_ creates an image of the BFP where an aperture is placed to block the reflected excitation light that comes with a large angle when collimated and that is focused off-axis by L_4_. After the aperture, another relay lens, L_5_, creates an image of the AuNPs on the camera. **b** Scheme of the TIR-based approach used to stabilize the focus. A focus displacement $\Delta z$ is translated into a lateral displacement $\Delta r$ of the focus signal in the image. **c** Image of the AuNPs scattered signal and of the focus reflection (red square). Detail of a single AuNP (indicated by a white square) and its 1D intensity profile (dots) fitted with a Gaussian function (red line), σ = 184 nm

A scheme of the optical setup for stabilization is shown in Fig. 1a. We used light in the near infrared (NIR) range between 750 and 830 nm. Then, the stabilization setup can be added as an additional module to a fluorescence microscope by including a suitable dichroic mirror (DM). The NIR beam was split in two (BS_1_) to serve both as the wide-field illumination for the fiducial markers and as the focused beam that will be reflected to track the axial drift.

The NIR beam used for Z-stabilization (focus-lock) was focused on the sample by the objective (upper excitation path in Fig. 1a). This beam was purposely shifted from the objective main axis to produce a tilt, so that an axial (Z) displacement of the sample with respect to the objective was revealed by a lateral translation of the reflection detected in a camera, as schematically shown in Fig. 1b. The NIR beam used for XY (lateral) stabilization (lower excitation path in Fig. 1a) was configured for wide-field illumination by focusing at the back focal plane (BFP) of the objective. Also in this case the beam was aligned off-center of the objective to achieve total internal reflection (TIR) illumination. The reflected light was blocked in the detection using an aperture. In this way, the light scattered by the AuNPs was detected with high signal-to-background ratio in a quasi dark-field mode. Both the reflected beam used to track the Z position and the image of the fiducials were acquired with the same camera. A representative image of the scattered signal from the fiducial markers and the reflected focus spot is shown in Fig. 1c, together with a zoomed-in view of a single fiducial and its Gaussian-fitted intensity profile. The sample was mounted on a XYZ piezo stage to compensate for the drift. Images of the actual optical setups are provided in the Supplementary Information (Figure S1).

### Control software architecture

**Fig. 2:**
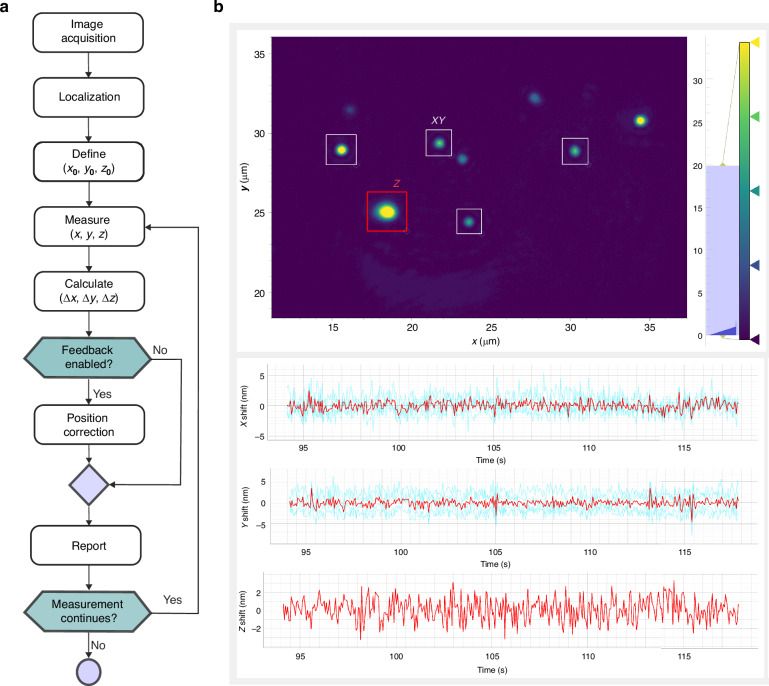
Stabilization system software. **a** Flowchart of the stabilization software indicating the main processes. **b** Screenshot of the main window of the graphical user interface. In the upper panel, real-time imaging of the focus beam (red square) and the selected ROIs for fiducial markers (white squares). In the lower panel, plots of individual (cyan lines for XY, red line for Z) and average displacements (red lines) over time

The stabilization software is designed to be easily integrated with other modules. It is freely available from https://github.com/Stefani-Lab/takyaq, and it is distributed under an OSI approved license (AGPL-3.0). The core of the stabilization system follows a loop of image acquisition, localization and position correction. A simplified flowchart of the process is shown in Fig. 2a. To initiate the stabilization a set of regions of interest (ROIs) is selected: one for each fiducial marker to be tracked (XY) and one for the focus (Z) reflection. The initial localization of the XY fiducial markers and Z beam reflection are defined as the setpoint positions $\left({x}_{0},{y}_{0},{z}_{0}\right)$. The stabilization process continuously acquires images and performs localizations of the signals on the selected ROIs. The position $\left(x,y\right)$ of each fiducial marker is estimated by fitting a 2D Gaussian profile on each ROI. For the focus signal, the (*z*) position is estimated using the center of mass of the reflected beam. Comparison of the determined current position $\left(x,y,z\right)$ with the setpoint $\left({x}_{0},{y}_{0},{z}_{0}\right)$ yields a displacement in 3D $\left(\Delta x,\Delta y,\Delta z\right)$. When more than one fiducial marker is used, the average lateral displacement $\left(\overline{\Delta x},\overline{\Delta y}\right)$ is calculated. If the stabilization system is enabled, a customizable response function is used to calculate the position correction. The software includes PI based response functions that performed well under our measurement conditions, as well as instructions and examples on how to develop custom response functions if necessary (See Supplementary Section “Software description”). Finally, the measured image and displacement are shown online after each acquisition, and the loop starts again. The XY and Z processing are completely decoupled, making it possible to use the software for either the lateral, axial or for both axes simultaneously.

The software includes a graphical user interface (GUI) that displays the image as well as the individual and average displacements (see Fig. 2b). The GUI provides simple means for easily selecting ROIs for XY fiducial markers and for the focus reflection as well as for starting/stopping the tracking and stabilization of XY and/or Z. The frontend also offers the possibility to set the PI feedback loop and camera parameters, and to send commands to the stabilization system to perform calibration of the camera pixel size and to move the stage to a determined position. The GUI allows users to save XY and Z displacement data for offline analysis.

The software is designed to be compatible with a wide range of cameras, piezo stages, and response functions without being dependent on specific hardware. This is achieved by interacting with generic camera and piezo objects that act as proxies between the stabilization system and the specific hardware. These objects must implement a small set of well-defined methods: a function for acquiring an image in the case of the camera, and functions to read the current position and to independently set the $\left(x,y\right)$ and ($z$) positions in the case of the stage. These abstraction layers allow the software to work with different hardware as well as to tune the response to different situations. For example, it supports the use of different correction strategies for different drift ranges and the ability to handle exceptional cases such as the detachment of a fiducial marker. These objects can optionally implement additional methods that enable access to extended functionality: define limits to the stage positions that can be set from the GUI, set the camera exposure time, etc. Depending on the specific setup, the X and Y directions of the camera may not coincide with the ones of the piezo stage, e.g. being either permuted or inverted. These differences must be compensated by the camera object, processing the image before returning it to the stabilization system.

The program is implemented in pure-python, and its dependencies are commonly used scientific libraries (numpy^32^ and scipy^33^). We provide a package for easy installation and a ready-to-use graphical frontend implemented in PyQt5, making it easy to integrate as-is with larger projects using the same framework. Graphics are handled using PyQtGraph library.

Our software also provides three practical features for nanoscopy applications. The first one is a procedure for the calibration of the camera pixel size and of the Z drift response using the piezo stage movement as a reference, which is key for a quantitative drift correction. The second is a mean for controlled shift of the reference positions, enabling stage movements under stabilized conditions, which are particularly useful for i) placing emitters over well-defined patterns for assessing the resolution of fluorescence nanoscopy, ii) re-center the excitation beam pattern of MINFLUX or RASTMIN over an emitter. The last one is the ability to save the absolute position of the focus, ensuring consistency even after sample changes. Details about these three features can be found in the Supplementary Information.

### System configuration and performance

**Fig. 3:**
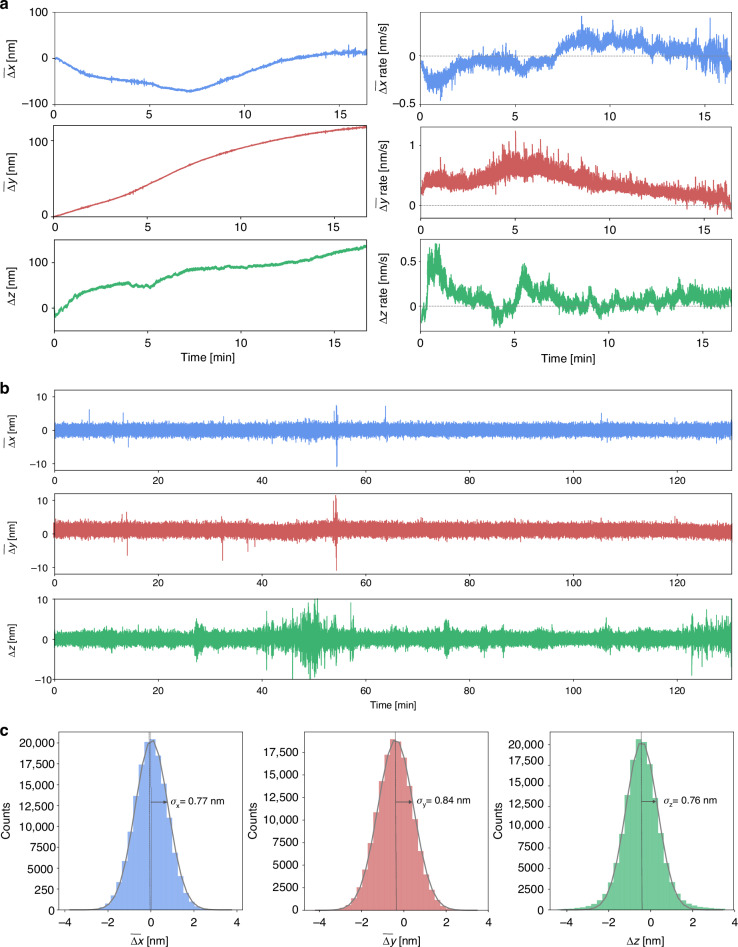
Performance of the stabilization system in the 3 dimensions (RASTMIN setup). **a** Sample position (left) and speed (right) in the X, Y and Z coordinates without an active stabilization. For X and Y, the average position obtained from 5 AuNPs was used. **b** Stabilized sample position as a function of time, using 4 AuNPs as fiducial markers for XY stabilization. **c** Distribution of the sample displacements in X, Y and Z coordinates. σ is the standard deviation obtained from a Gaussian fit (grey lines)

Sample drift may occur at a wide range of time-scales, depending on each experimental setup, level of mechanical and acoustic isolation, and sources of noise, vibrations and air currents, among other factors. In our case, and most microscopes evaluated in the literature, the fastest sample drift speeds are in the order of 1 nm/s (Fig. 3a). Therefore, achieving sub-nm stabilization requires measurements and correction of the sample position with sub-nm precision in less than 1 s. The overall processing time of the software and the stage’s response time are of a few ms. Thus, response time of the stabilization system is mainly limited by the image acquisition time and the communication speed between the PC and the piezo stage controller.

The minimum image acquisition time is determined by the signal quality required to achieve sub-nm localization precision of the fiducials and of the reflected beam, which in turn depends on fiducial marker brightness, laser power and camera sensitivity. In both setups, with the 200 nm AuNP fiducials, and a NIR laser power of 0.3 mW (at the back focal plane of the objective), an exposure time of 50 ms delivered suitable fiducial images. Regarding cameras, they usually have the option to operate in triggered mode (the image is acquired on request) or in freerun mode (images are acquired continuously). Although freerun mode allows to achieve higher correction frequencies, we found more convenient to use the triggered mode because otherwise the piezo position may change during image acquisition.

The sample position correction rate depends on the communication between the PC and the piezo controller. In the MINFLUX setup the stabilization module is loaded as part of a larger Python program that controls the microscope, therefore the CPU load is shared between different tasks of the same process. Under these conditions, the average correction period (i.e. total time needed to perform a sample position measurement and correction) was ~92 ms. In the RASTMIN setup, the stabilization runs as a standalone program leading to a faster average correction period of ~54 ms. Since the stabilization system is itself a closed loop system, the piezo stage controller was operated in open-loop mode to achieve a faster response. We noted that operating the piezo controller in closed-loop introduced an additional lag. The minimum step that can be performed by the stage (its resolution) depends on the bit depth of the stage driver and its travel range. In our case, with a 15-bit dynamic range for a 20 µm travel, the minimum stage step is 0.61 nm, defining a lower bound for position corrections.

The software is designed to be fast and provide an almost continuous drift correction. To minimize the analysis time, the previous localization values are used as seeds for fitting the new localizations, and fitting is performed in parallel using multiple processes. The total overhead of the analysis is in the range of a few ms.

While, in principle, a single fiducial marker is sufficient for XY stabilization, it is advantageous to use more and compute the average lateral displacement. First, if any given selected marker performs poorly (e.g., it is loosely bound to the surface, it scatters less number of photons or it is located on a region with high background), its effect is diluted when averaging the displacements of several markers. Second, the stabilization precision calculated from the average displacement of *K* nanoparticles is $\sqrt{K}$ times better than in the case of using a single particle. In practice, the number of usable markers is limited by the density of fiducial markers in the sample, which cannot be indefinitely high because it can interfere with the sample imaging. Moreover, a high density of fiducial markers increases the likelihood of one of them being superposed with the beam used for focus locking, thereby impairing axial stabilization.

To evaluate the performance of the stabilization system we recorded time series of $\left(\Delta x,\Delta y,\Delta z\right)$ in two different setups: p-MINFLUX and RASTMIN. In Fig. 3 we show an example of performance obtained in the RASTMIN setup (results for the p-MINFLUX setup are shown in Figures S2–4). Figure 3a shows the sample drift in the absence of stabilization in our RASTMIN setup, with the average lateral position obtained from 5 AuNPs and the axial position measured from the coverslip reflection. When only Z-stabilization is active, lateral drift remains significant in the absence of XY correction (see Figure S3). Figure 3b displays the average lateral displacement of the selected 4 AuNPs $\left(\bar{\triangle x},\bar{\triangle y}\right)$ and the axial displacement $\Delta z$ versus time when the sample is stabilized, for 130 minutes, using a camera exposure time of 50 ms. Figure 3c shows the histograms of displacements fitted with a Gaussian function to estimate their standard deviation around the setpoint value, obtaining *σ*_*x*_ = 0.77 nm, *σ*_*y*_ = 0.84 nm and *σ*_*z*_ = 0.76 nm. The achieved stabilization is better than 1 nm in all 3 dimensions for more than 2 hours.

### RASTMIN and p-MINFLUX measurements

**Fig. 4:**
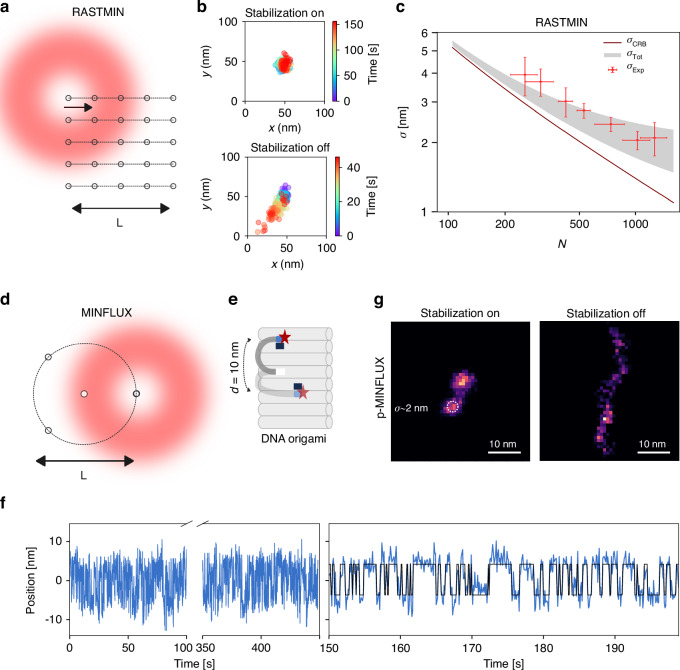
Localization precision achieved with the stabilization system in RASTMIN and imaging of a dynamic DNA origami structure with p-MINFLUX. **a** Schematic of the excitation beam pattern for RASTMIN. **b** Comparison of the performance of RASTMIN to localize a single-molecule with and without active stabilization. Sample: DNA origami structure with a fixed single ATTO 647 N fluorophore. Measurement time: 150 s (216 ms time bins, $\bar{N}=1000$). Without the stabilization, the effective acquisition time was limited to ~40 s due to sample drift causing the fluorophore to move outside the excitation beam pattern. **c** Localization precision (*σ*_*Exp*_) for RASTMIN measurements of single ATTO 647 N molecules with different photon counts (N). For each N, four independent measurements were performed in which 100 localizations were obtained. The theoretical lower bound for precision (*σ*_*CRB*_) is included for comparison (solid line). The expected precision (*σ*_*Tot*_) taking into account the stabilization ($1{\rm{nm}} < {\sigma }_{{Stabilization}} < 2\,{\rm{nm}}$ in this case) is also shown (gray shaded area). **d** Schematic of the excitation beam pattern for *p*-MINFLUX. **e** Schematic of the dynamic DNA origami “clock” structure with a single ATTO 643 fluorophore and two docking sites separated 10 nm from each other. **f** 450 s localization trace (binning time = 100 ms) along the main axis of the DNA origami using p-MINFLUX and 50 s detail of the trace. **g** Comparison of the performance of p-MINFLUX with and without active stabilization. Sample: dynamic DNA origami “clock” where a single ATTO 643 fluorophore switches between two positions separated by 10 nm. Measurement time: 112 s (70 ms time bins, $\bar{N}=1800$)

As a demonstration of the key importance of stabilizing the sample with (sub)-nm precision, we showcase RASTMIN and p-MINFLUX measurements. For RASTMIN (Fig. 4a), we measured single molecules (ATTO 647 N) attached to DNA origami structures that were immobilized on a glass surface through neutravidin-biotin chemistry. We used a fixed frame rate of 100 ms per frame and varied the laser power to attain different photon count rates. We show in Fig. 4b the performance of RASTMIN to localize a single-molecule with and without active stabilization. The experimental localization precision (*σ*_*Exp*_) was then calculated for time windows of 10 seconds as the standard deviation of the 100 localizations obtained within that time frame (see Methods for further details). In Fig. 4c, we show the comparison of *σ*_*Exp*_ for different number of photons N along with the Cramer-Rao Bound *σ*_*CRB*_ (theoretical maximum precision that can be achieved for our position estimator)^26^ calculated using the experimental PSFs. The experimental precision matches the expected precision considering *σ*_*Total*_ = (*σ*^*2*^_*CRB*_ + *σ*^*2*^_*Stabilization*_)^½^, with *σ*^*2*^_*Stabilization*_ = *σ*^*2*^_*x*_ + *σ*^*2*^_*y*_ the average lateral stabilization precision, reaching a value of *σ*_*Total*_ = 2-3 nm with only 500–1000 photons.

Next, we performed p-MINFLUX (Fig. 4d) measurements of a dynamic DNA origami structure, where a protruding single-stranded DNA (ssDNA) functionalized with a fluorescent dye (ATTO 643) at the end is able to transiently bind to two docking strands that are separated 10 nm one from the other, as depicted in Fig. 4e. When the stabilization is turned on and working with ~ 1 nm precision, the dynamic transitions between the two docking sites of the DNA origami are clearly observed for over 8 min (Fig. 4f), demonstrating that our system can be used for long acquisitions and still guarantee nanometer-scale resolution. Another example is shown in Fig. 4g, where we show 2D histograms of the positions recorded with (left) and without (right) the stabilization system on. Only with the stabilization system the two docking sites of the DNA origami are fully resolved with widths in agreement with σ_CRB_ convoluted with the stabilization precision (2.1 nm experimental precision vs. 1.9 nm of *σ*_*Total*_). We note that offline drift correction using the recorded positions of the AuNPs is not sufficient to reconstruct the DNA origami docking sties. This is because during the measurement time the DNA origami drifts away from the center of the excitation beam pattern of p-MINFLUX, resulting in poorer localization precisions (Figure S5). For these type of measurements, i.e. nano-imaging or tracking within the excitation beam pattern, the function of the stabilization system is to keep the sample position stable. By contrast, for single-molecule or single-particle tracking over regions larger than the excitation beam pattern, the stabilization system should be included in an additional control loop to periodically recenter the target molecule/particle in the excitation beam pattern.

## Discussion

To date, nanometer-scale techniques like MINFLUX have had tremendous impact in visualizing and tracking proteins with unprecedented details, reaching nanometer resolution at milliseconds timescales. However, the access to these technologies has remained limited to a few groups with high expertise in optics and instrumentation or with access to very expensive commercial setups. In these methods, the relative position of the sample with respect to the excitation beam pattern is critical to achieve nanometer precision. Therefore, they require an active stabilization of the relative position of the sample and the objective, with the highest possible precision during the relatively long measurement times.

We have developed a system and open-source software for active stabilization of an optical microscope that delivers a precision below 1 nm in three dimensions over hours. The optical setup is simple and can be added to a fluorescence microscope as an independent separate module. We showcased its modularity and performance by localizing single molecules with 1–3 nm precision and resolving 10 nm distances in DNA origami structures in two different fluorescence nanoscopes, a RASTMIN and a pulsed-interleaved MINFLUX.

Because a single camera is used for both lateral and axial tracking, our method only needs a single image to achieve stabilization in the three dimensions, which facilitates its implementation in terms of hardware and software. All operations required for the stabilization are performed relatively fast (in the order of 50 ms) and run perfectly on a standard CPU. The open-source Python software we provide can be adapted to different hardware with minimum effort. Additionally, the system offers the possibility to track/correct only the lateral drift, only the axial drift, or all directions, giving it versatility to be employed in different imaging techniques (e.g., SMLM widefield methods like DNA-PAINT or STORM, as well as scanning modalities like STED), and different types of samples; e.g., with and without fiducial markers.

The stabilization system presented here with a simple and affordable experimental design, together with the modularity and user-friendly open-source software will facilitate that more research groups build and apply ultraprecise single-molecule localization methods like MINFLUX, p-MINFLUX or RASTMIN, making these powerful tools for discovery more accessible and increasing their impact in fields such as materials science, nanophotonics, and chemistry, among others.

## Materials and Methods

### Sample preparation

#### PSF sample and performance sample

1.5 thickness coverglass with CoverWell™ Imaging Chambers (Grace Bio-Labs) were treated with 2% Hellmanex^TM^ for 30 min, washed with Milli-Q water and functionalized with a positively charged layer of Poly diallyldimethylammonium chloride (PDDA) Mw = 400,000 − 500,000 (Sigma-Aldrich), by incubating for 30 min a solution of PDDA (1 mg/mL in 0.5 M NaCl). Next, the chamber was rinsed (3×) with Milli-Q water and incubated for 10 min with a solution (1:50 from original 1 OD stock) containing 200 nm spherical gold nanoparticles (Nanopartz) diluted in PBS 1X (12.5 mM). Next, the chamber was rinsed (3×) with PBS 1X (12.5 mM) and incubated for 10 min with 40 nm Dark Red FluoSpheres Fluorescent Microspheres (Thermo Fisher Scientific). The beads solution was prepared directly by diluting the stock solution in PBS 1X (12.5 mM) (10^−^^6^ dilution from original stock) and was sonicated for 10 min just before being added to the chamber.

#### DNA origami samples

1.5 thickness coverglass with CoverWell™ Imaging Chambers (Grace Bio-Labs) were treated with 2% Hellmanex^TM^ for 30 min, washed (3×) with Milli-Q water and functionalized with BSA-biotin (1 mg/mL, 15 min incubation). Next, the chamber was washed (3×) with Milli-Q water and incubated for 15 min with Neutravidin (1 mg/mL). After washing (3×) with Milli-Q water, a solution containing 200 nm spherical gold nanoparticles (Nanopartz) diluted (1:50 from original 1 OD stock) in PBS 1X + MgCl_2_ (12.5 mM) was incubated for 10 min. Next, the chamber was rinsed (3×) with PBS 1X + MgCl_2_ (12.5 mM) and incubated for 10 min with DNA origami samples in PBS 1X + MgCl_2_ (12.5 mM) (DNA origami concentration = 50 pM). Next, chambers were washed (5×) with PBS 1X + MgCl_2_ (12.5 mM) and filled with imaging buffer (TAE 1x –40 mM Tris, 20 mM acetic acid, 1 mM EDTA–, 2 mM Trolox/Troloxquinone, 12.5 mM MgCl_2_, 3% glycerol, 1.25 mM KCl, 1% (w/v) D-(+)-glucose, 0.6 mg/mL glucose oxidase –Sigma-Aldrich– and 50 μg/mL catalase –Sigma-Aldrich–).

### p-MINFLUX setup

The setup used for p-MINFLUX is essentially the one described in ref. ^5^. Briefly, the excitation is a pulsed supercontinuum laser (SuperK EVO, NKT Photonics) with a spectral range of 400–2400 nm and a repetition rate *f* = 20 MHz (*T* = 50 ns), and a pointing accuracy of 0.9 mrad. The excitation is divided into four beams that are coupled into optical fibers of different lengths to introduce time delays $\Delta t={\rm{T}}{\rm{ / }}4$ between the beams. After out-coupling of the fibers, the beams are recombined and passed through a 0 − 2*π* vortex phase plate (V-633-10, Vortex Photonics) and polarization optics to create doughnut-shaped foci at the sample with a high NA objective (CFI Plan Fluor 100x, NA = 1.4, Nikon Instruments Inc.). The sample is mounted on a piezoelectric stage (NanoMax MAX311D/M with controller BPC303, Thorlabs). Each beam path has independent mirrors equipped with piezo motors (PIAK10, Thorlabs) for precise alignment of the four donut-shaped foci in the MINFLUX excitation pattern. Fluorescence from the sample is spectrally selected using dichroic beam splitters (ZT532/640rpc-UF, Chroma Technology Corp) and band-pass filters (ET705/72 M, Chroma Technology Corp and NF808-34, Thorlabs), before being detected by an avalanche photodiode APD (SPCM-AQR-13, PerkinElmer Optoelectronics). Photon counts from the APD are read out with a time tagger device (Time Tagger Ultra, Swabian Instruments).

For the stabilization system (Fig. 1a), a near-infrared band from the white laser is selected with a bandpass filter (ET808/20, Thorlabs). A pellicle beamsplitter (8:92, R:T, BP108, Thorlabs) directs a fraction of this beam for axial stabilization, while the main beam, expanded by a telescope system composed of lenses L_2_ and L_3_ (*f*_*L*2_ = 30 mm, *f*_*L3*_ = 200 mm), provides lateral stabilization via widefield illumination at the sample plane. In the axial path, the lens L_1_ (*f*_*L*1_ = 200 mm) is matched with the tube lens (TL) of the microscope in a *4* *f* system. Both beams are combined using a beamsplitter cube (CCM1-BS013/M, Thorlabs), pass through the TL (*f*_*TL*_ = 250 mm), and are coupled into the main optical path with a dichroic mirror (T750SPXRXT-UF1, Chroma Technology Corp). The beam for Z-correction is reflected at the sample interface and directed by a beamsplitter (BP145B1 R45% T55%, Thorlabs) towards a CMOS camera (U3-3060CP-M-GL, IDS), where it is focused along with the light scattered by the fiducial particles. A notch filter (ZET647NF, Chroma Technology Corp) is used to block residual excitation light from reaching the camera. Lenses L_4_ and L_5_ (*f*_*L*4_ = 100 mm, *f*_*L5*_ = 75 mm) form the image of the AuNPs and the reflected beam in the camera. All lenses used in this setup, except for the objective lens, are achromatic doublets from Thorlabs.

### RASTMIN setup

The RASTMIN technique was implemented in a custom-made confocal microscope with a beam-scanner based on galvo mirrors and a piezoelectric stage to control the motion of the sample holder (a complete description of the setup can be found in ref.^6^). Excitation is performed using a linearly polarized pulsed laser at 640 nm (200 ps pulse width, PicoQuant LDH-P-C-640B) operating at 40 MHz repetition rate. A Glan-Thomson polarizer (Thorlabs) and a half-wave plate (AQWP05M-600, Thorlabs) are employed to control the polarization tilt as the beam is directed towards a spatial light modulator (SLM-100, Santec) to generate the doughnut-shaped focus. Subsequently, circular polarization is adjusted using a half-wave plate and a quarter-wave plate. The SLM is controlled using open-source Python code^34^. The beam is then directed to the scanner system, which consists of two lenses, two orthogonal galvanometric mirrors (6215H, Cambridge Technology) (horizontal, x, and vertical, y), and one concave mirror. The voltages required to drive the galvanometric mirrors are provided by a linear-regulated power supply (Peaktech 6060) controlled with a DAQ board (PCIe-6353, National Instruments). The DAQ board is in turn interfaced via the specialized software Imspector. Next, light is collimated by the tube lens of the microscope and focused into the sample with an objective with 1.4 NA (Leica HCX PL APO 100x/1.40-0.70 Oil CS). The sample is mounted on a piezoelectric stage (NanoMax MAX311D/M with controller BPC303, Thorlabs). The fluorescence emission arising from the sample passes through the same galvo-based scanning system described above, then through a long pass dichroic mirror (FF649-Di01-25 × 36, Semrock), an emission band-pass filter (ET700/75 m, Chroma Technology Corp) and is finally focused into an avalanche photodiode detector (SPCM-AQR-13, PerkinElmer Optoelectronics). Two notch filters (ZET647NF and HQ655LP, Chroma Technology Corp) are placed in the emission path to avoid detecting back-reflections of the excitation beam.

The stabilization system is added to the microscope by combining the excitation/fluorescence light with an IR laser (830 nm laser, Lambda mini EVO rgb-lasersystems, pointing accuracy < 1 mrad) through a dichroic mirror (T790SPXRXT, Chroma Technology Corp). The paths (Z focus path and XY stabilization path) are divided by a 50:50 polarizing beam-splitter (CCM1-PBS251/M, Thorlabs). In the axial path, the lens L_1_ (*f*_*L*1_ = 200 mm) is matched with the tube lens (TL) of the microscope in a *4* *f* system. In the XY path, the telescope formed by L_2_ and L_3_ (*f*_*L*2_ = 30 mm, *f*_*L3*_ = 300 mm) expands the beam which is focused on the back focal plane (BFP) of the objective by the TL (Leica, *f*_*TL*_ = 200 mm). Both excitation paths are combined by a Polarizing Cube Beamsplitter (10FC16PB.5, Newport). A Pellicle Beamsplitter (BP145B1, Thorlabs) is used to separate excitation light from scattered/reflected light, which is the signal that will be detected on the camera (UI-3060CP-M-GL Rev 2, IDS). We also include an aperture in the detection path to block the reflected wide field illumination light that is focused off-axis. All lenses used in this setup, except for the objective lens and the tube lens, are achromatic doublets from Thorlabs.

### p-MINFLUX and RASTMIN measurements and data analysis

p-MINFLUX and RASTMIN localizations were obtained as previously described in refs. ^5,6^. For RASTMIN, PSFs were obtained by acquiring 16 images of a single fluorescent bead (pixel size = 6 nm, scanned area of 1.2 × 1.2 μm^2^), maintaining the stabilization on, and averaging all the acquired images. In p-MINFLUX, PSFs were obtained by scanning an area of roughly 400 ×400 nm^2^ with the four donuts turned on at the same time and only Z stabilization (the piezo stage is used for scanning). Time-gated photons were used to reconstruct each individual PSF. The background signal, necessary for calculating the signal-to-background ratio (SBR), which is in turn needed to estimate the single emitter position and compute the CRB, was measured in both techniques by analyzing the number of photons detected after the photobleaching of single molecules, for each laser power used (we obtained SBR = 3 for RASTMIN and 6 for p-MINFLUX using the highest laser power).

To estimate the localization precision (*σ*_*Exp*_) for RASTMIN, traces were acquired varying the laser power (from 6 to 50 μW, measured at the back focal plane of the objective) in order to achieve a different number of photons for a fixed frame time of 100 ms. The reported precisions were calculated as the average standard deviation of localizations in both the x and y directions within ten-second time windows. Each point on the plot of Fig. 4c represents the mean precision of four different dyes, with error bars indicating the standard deviation of the precision (y-axis) and the number of photons (x-axis). The CRB curve (*σ*_*CRB*_) used for comparison was obtained as the average CRB from a 10 × 10 nm^2^ square area that contained the positions measured experimentally. For each point on the curve, a fixed N value was used, and the background was calculated as the sum of two components: dark counts, which are independent of laser power and remain around 300–400 counts per second in our setup, and a background component that increases linearly with laser power. As a result, points on the curve with low signal (corresponding to low laser power, where dark counts contribute a larger fraction of the background signal) exhibit a slightly lower SBR compared to points with high signal.

The p-MINFLUX time trace obtained using the dynamic DNA origami structure and with the stabilization system turned on was analyzed as follows: i) time binning was fixed to 100 ms, in order to have roughly 1700 photons per bin, ensuring ~ 2 nm localization precision; ii) a main axis of the origami was obtained from a Singular Value Decomposition of the localizations, and the localizations were then projected into this axis; iii) a HMM (hidden Markov model) analysis was performed to the trace using the projected coordinates into the main axis; and iv) from the HMM analysis, transition bins were filtered out (i.e. the first and last frame of each state) before plotting the 1D and 2D histograms. The reported localization precision of 2 nm was obtained using a Gaussian mixture model (two 2D Gaussians) of the curated dataset (i.e. after filtering the transition bins). Regarding the data obtained without stabilization, post-acquisition drift correction was performed using the average displacements obtained from the trajectories of several fiducial markers. To match the time of both acquisitions (p-MINFLUX and AuNPs tracking), we used as reference the initial time of both files.

### DNA origami structures design

Staples used to fold the two origamis are shown in Tables S1 and S2.

## Supplementary information

Supplementary Information for Open-source Sub-Nanometer Stabilization System for Super-resolution Fluorescence Microscopy

## Data Availability

Data is available from the corresponding author upon request.
