# Supplementary material for: Open-source sub-nanometer stabilization system for super-resolution fluorescence microscopy
Source: Light Sci Appl. 2025 Nov 20;14:385. doi: 10.1038/s41377-025-02022-6 (PMC12630732; doi:10.1038/s41377-025-02022-6)
Supplement: Supplementary file 1 — Supplementary Information for Open-source Sub-Nanometer Stabilization System for Super-resolution Fluorescence Microscopy [file 41377_2025_2022_MOESM1_ESM.docx]

**Supplementary Information for**

**Open-source Sub-Nanometer Stabilization System for Super-resolution Fluorescence Microscopy**

Florencia Edorna,^1,2,#^ Florencia D. Choque,^1,2,#^ Giovanni Ferrari,^1,3^ Luciano A. Masullo^1§^, Piotr Zdańkowski,^4^ Guillermo P. Acuna,^5,6^ Philip Tinnefeld,^3^ Alan M. Szalai,^1^ Lucía F. Lopez, ^1^* Andrés Zelcer, ^1^* and Fernando D. Stefani^1,2^

^1^ Centro de Investigaciones en Bionanociencias (CIBION), Consejo Nacional de Investigaciones Científicas y Técnicas (CONICET), Godoy Cruz 2390, C1425FQD Ciudad Autónoma de Buenos Aires, Argentina.

^2^ Departamento de Física, Facultad de Ciencias Exactas y Naturales, Universidad de Buenos Aires, Güiraldes 2620, C1428EHA Ciudad Autónoma de Buenos Aires, Argentina.

^3^ Department of Chemistry and Center for NanoScience, Ludwig-Maximilians-Universität München, Munich, Germany, Butenandtstraße 13, 81377.

^4^ Warsaw University of Technology, Institute of Micromechanics and Photonics, A. Boboli 8, 02-525 Warsaw, Poland

^5^ Department of Physics, University of Fribourg, Chemin du Musée 3, Fribourg, CH-1700, Switzerland

^6^ Swiss National Center for Competence in Research (NCCR) Bio-inspired Materials, University of Fribourg, Chemin des Verdiers 4, CH-1700 Fribourg, Switzerland.

^#^ These authors contributed equally to this work.

* Corresponding authors: lucia.lopez@conicet.gov.ar, andres.zelcer@cibion.conicet.gov.ar

^§^ Present address: Max Planck Institute of Biochemistry, Am Klopferspitz 18, 82152 Planegg, Germany

Table of Contents

[Supplementary Text: Software description 2](#_Toc201912900)

[Supplementary Figures 6](#_Toc201912901)

[Figure S1. Images of the actual optical setups 6](#_Toc201912902)

[Figure S2. Sample X, Y and Z drifts of p-MINFLUX setup (non-stabilized). 7](#_Toc201912903)

[Figure S3. Sample X, Y and Z drifts of p-MINFLUX setup (Z stabilization only). 8](#_Toc201912904)

[Figure S4. Performance of the stabilization system in the 3 dimensions for the p-MINFLUX setup. 9](#_Toc201912905)

[Figure S5. p-MINFLUX measurement with no active stabilization and post processing drift correction. 10](#_Toc201912906)

[Supplementary Tables 10](#_Toc201912907)

# Supplementary Text: Software description

*Requirements and technical details*

The stabilization system only requires NumPy and SciPy packages. To use the provided GUI, PyQT5 and pyqtgraph are also required. We keep supporting Python from 3.7 onwards, as there are a lot of legacy programs that haven’t been updated to modern versions yet.

The stabilization loop runs on its own thread. The reference Python implementation (CPython) uses a Global Interpreter Lock (GIL) that makes it very easy for one thread of the same process to keep others from running. Therefore, care must be taken not to hog the CPU running large portions of Python code in another thread of the same process. Some libraries (notably numpy) release the GIL before performing many operations and will not interfere with the stabilization. As usual, I/O operations also release the GIL.

*Application Program Interface (API)*

The software provides a well-defined Application Program Interface (API) that controls the stabilization module. The provided GUI accesses this API to perform all required actions. Therefore, there is no need to directly access the API unless you want to implement a different GUI or use the stabilization software without one. The API provides methods for setting the ROIs, starting and stopping tracking and stabilization, initiating calibrations, adjusting the reference position, and adding callbacks for events like stabilization start and stop, and for each localization/tracking/correction cycle. See the documentation provided on the repository (https://github.com/Stefani-Lab/takyaq) for full description of the API. The main distinct features that are included are:

Calibration

To calibrate the pixel size of our imaging system, a reference sample can be used. In our laboratory, we use a high-quality reference grid sample (Thorlabs R1L3S3P). Our stabilization software offers a different, complementary possibility for calibration: using the piezo stage movement as the distance reference. The software will perform a series of controlled stage movements and localizations, reporting the results. In the case of X and Y calibration, the pixel size is reported.

In the case of the focus signal, one must always rely on the piezo movement as the distance reference. Depending on the relative position and angle of the focus beam with respect to the optical axis, the amount and direction of displacement of the reflection will vary. The procedure provided for Z calibration reports both values as the shift in nm per pixel and the direction as the angle from the +X direction. Therefore, the Z drift is determined using both the magnitude and direction of the reflected beam position shift. In this way, a maximum sensitivity is obtained as all available data is used.

Reference shift

Testing the performance of a super-resolution microscope is fundamental. A common procedure to test the resolving power is measuring the location of the same entity after performing small position shifts. In order to perform these movements keeping the precision provided by the stabilization, the software provides an instruction to shift the stabilization reference position while the stabilization is active, effectively forcing the stabilization system to move the stage. This feature provides a simple mean to execute predefined patterns (e.g. square patterns with 20 nm sides).

Z-lock saving

Many techniques rely on structured illumination, with well-defined PSFs’ positions and shapes. Actually, in many cases like 2D MINFLUX or RASTMIN what is relevant is the PSF XY profile at the focus position. As small changes in the focus can lead to changes and shifts in the XY profile of the PSF, it is therefore necessary to ensure that the same axial position is used both for PSF characterization and for the actual measurements. Otherwise, using misleading PSFs’ profiles and/or relative positions within the excitation pattern would lead to undesired biased localizations. The program provides a function to save all relevant data (ROI, Z reflection center of mass position in the image) to perfectly recover the same focus position.

PI parameters tuning

Adjustment of PI parameters must be performed after pixel size calibration. Set the period to 0.050 s and manually adjust the focus. Adjust the laser intensity, and camera exposure time, and optionally gain, to maximize the signal intensity of the fiducials and of the Z reflection without saturating the camera. Set both *Kp* and *Ki* to 0 for all axes. Let the program run with tracking enabled and correction disabled for a couple of minutes to have an idea of the setup stability. As XY stabilization depends on having a correct focus, XY is also lost when Z drifts away. Therefore, Z adjustments must be performed first.

Engage Z correction only, set Z *Kp* to 0.1 and and wait ~60 s between adjustments to acquire enough data to have proper statistics. Data can be reset after changing *Kp* using the “Clear” button. Check the position dispersion (‘σZ’, under the “Live Statistics” box). Increase the *Kp* value in steps of 0.1, clearing the data after each adjustment and waiting to acquire enough data to have stable statistics. Stop increasing *Kp* when the stabilization stalls or worsens, go back to the previous *Kp* value and repeat the procedure using steps of 0.05. The change in performance when using good *Kp* values is quite flat, so a finer tuning is not necessary.

Engage XY correction and repeat the procedure used for the Z axis successively for the X and Y axis.

Once *Kp* values for all axes have been found, the same procedure can be used to find the optimal *Ki*. Nevertheless, in this case it is necessary to acquire more data to observe an effect (~120 s). As the system applies corrections at a high rate, the effect of *Ki* is usually marginal and good results are obtained with *Ki* set to 0.

Implementing response functions.

The response functions to correct the sample position are implemented as methods of “Controller” objects. The file base_classes.py provides an abstract base class that can be used as a base to ensure consistency. A controller object must expose three methods:

reset_xy: This method is called when XY stabilization is engaged. It is called with the number of XY ROIs (as an int) as its only parameter. The controller should initialize or reset its internal data, preparing for beginning stabilization on the XY axes.

reset_z : This method is called when Z stabilization is engaged. It is called without parameters. The controller should initialize or reset its internal data, preparing for beginning stabilization on the Z axis.

response: This method is called on each stabilization cycle. it should return a 3-item tuple representing the response in X, Y and Z. Please note that a 3-tuple must be returned even if any stabilization is disabled.

It receives 3 parameters:

- A float t containing the timestamp of the localizations. It is the time as provided by Python’s time.time() call.
- xy_shifts, an array of shape (n_ROIS, 2). Each item of the array is the measured X and Y shift for each of the XY ROIS. If the localization of a particle fails (for example when the fitting procedure does not converge), the corresponding shifts will be numpy.nan, therefore the implementations must properly handle nan values. If XY stabilization is disabled, this parameter is None.
- z_shift: The shift on the Z axis as a float.

Note that the frontend provided is designed around the provided PI controller, and therefore expects the controllers to expose two extra methods: set_Kp and set_Ki. Both methods receive either a single float or a collection (tuple, list or array) of three floats. These methods are called when the corresponding values are set. If a single value is received, the same value should be used for all the axes. If a collection is received, the elements correspond to the value of the constant for the X, Y and Z  in order.

# Supplementary Figures


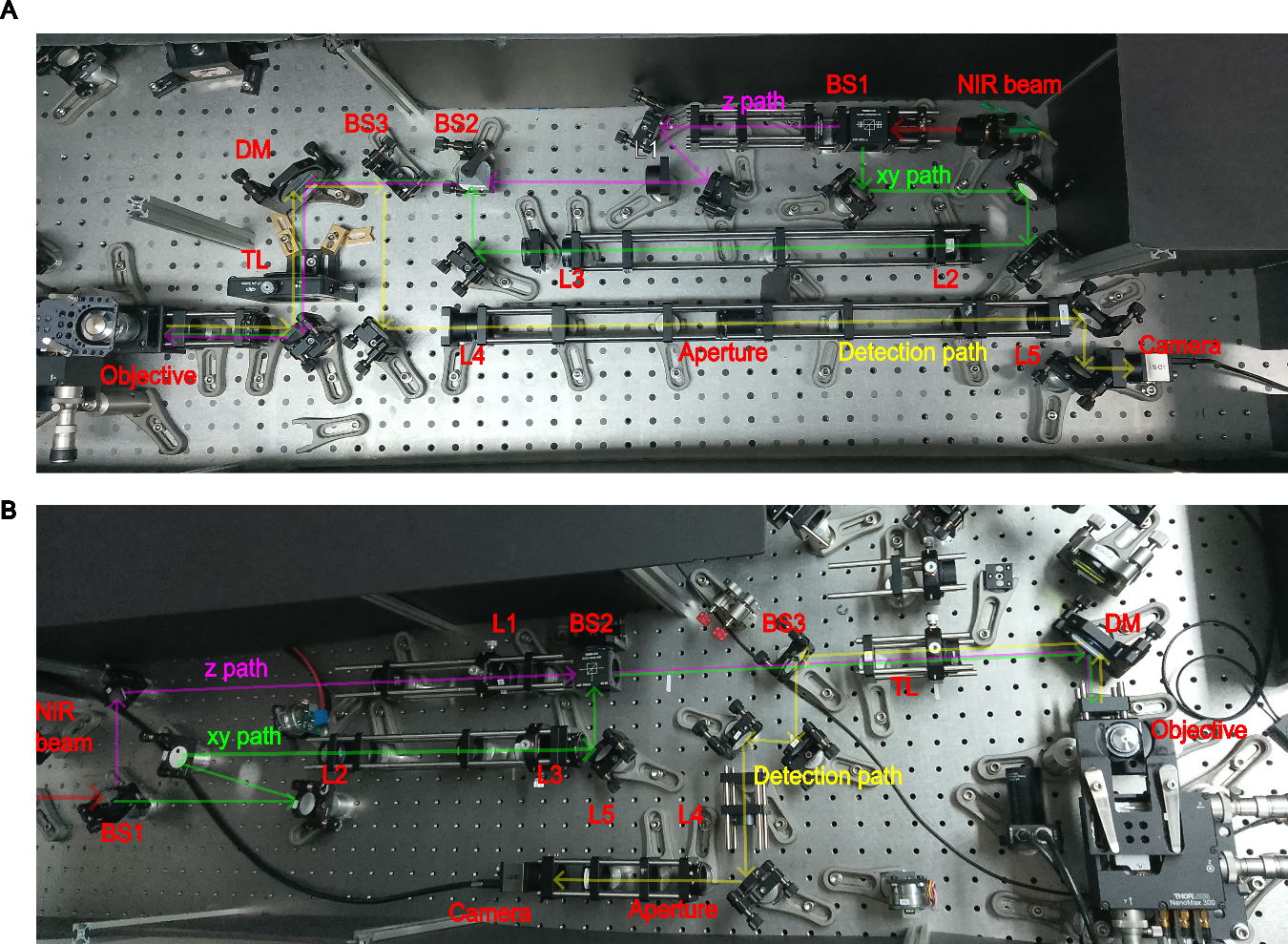


Figure S1. Images of the actual optical setups. (A) Stabilization system in the RASTMIN setup. List of the optical components: NIR beam: 830nm laser, Lambda mini EVO rgb-lasersystems; BS1: CCM1-PBS251/M, Thorlabs; BS2: 10FC16PB.5, Newport; BS3: BP145B1, Thorlabs; DM: T790SPXRXT, Chroma Technology Corp; L1:  f = 200 mm, Edmund; L2:  f = 30 mm, Edmund; L3: AC254-300-B-ML, f = 300 mm, Thorlabs; L4: AC254-200-B-ML, f = 200 mm, Thorlabs; L5: AC254-150-B-ML, f = 150 mm, Thorlabs.; TL: f = 200 mm, Leica; Objective: HCX PL APO 100x/1.40-0.70 Oil CS, Leica; Camera: UI-3060CP-M-GL Rev 2, IDS. (B) Stabilization system in the MINFLUX setup. List of the optical components: NIR beam: SuperK EVO, NKT Photonics; BS1: 8:92, R:T, BP108, Thorlabs; BS2: CCM1-BS013/M, Thorlabs; BS3: BP145B1 R45% T55%, Thorlabs; DM: T750SPXRXT-UF1, Chroma Technology Corp; L1: AC254-200-B-ML, f = 200 mm, Thorlabs; L2: AC254-030-B-ML, f = 30 mm, Thorlabs; L3: AC254-200-B-ML, f = 200 mm, Thorlabs; L4: AC254-100-B-ML, f = 100 mm, Thorlabs; L5: AC254-075-B-ML, f = 75 mm, Thorlabs; TL: AC254-250-B-ML, f = 250 mm, Thorlabs; Objective: CFI Plan Fluor 100x, NA = 1.4, Nikon Instruments Inc; Camera: U3-3060CP-M-GL, IDS.

**
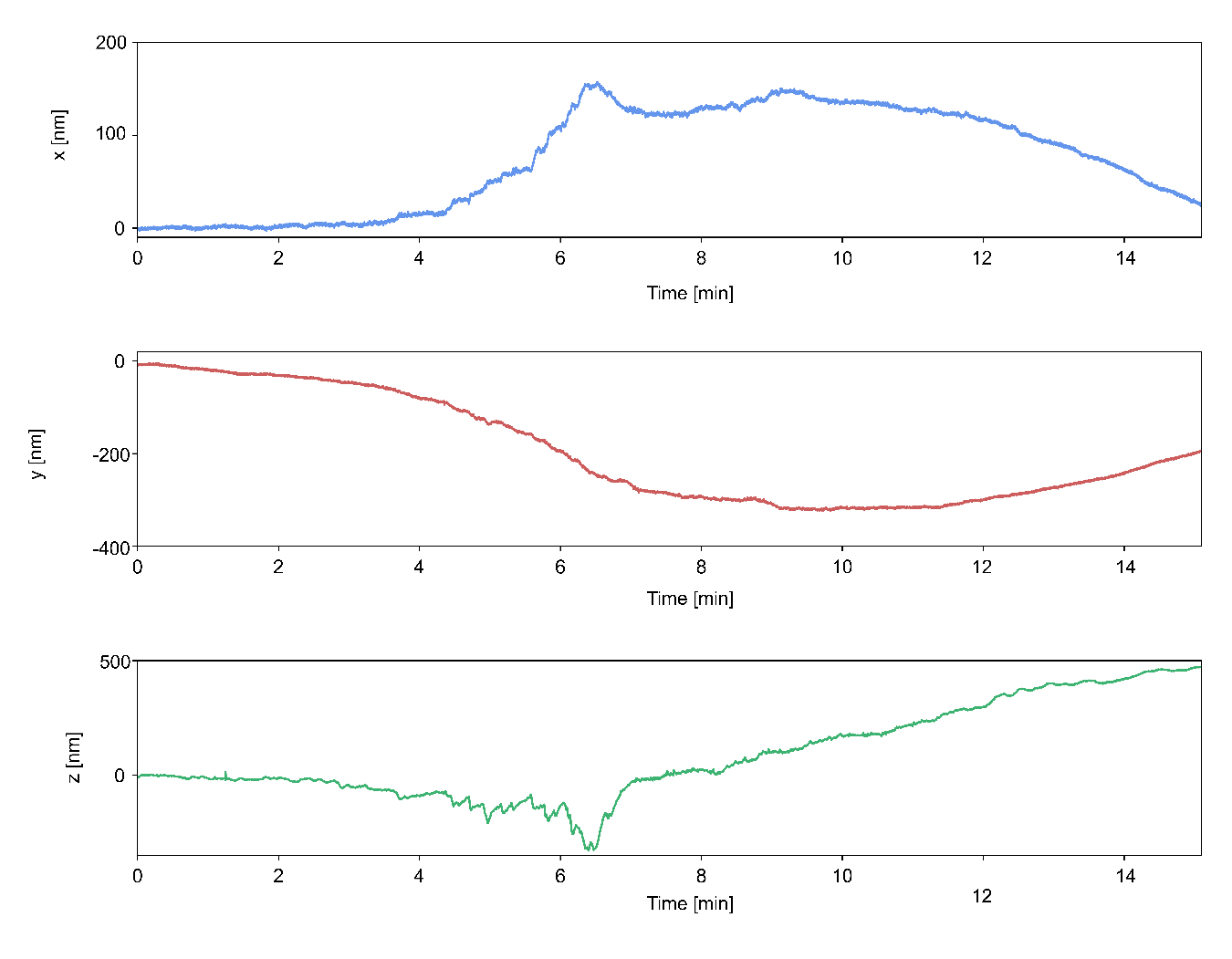
**

Figure S2. Sample X, Y and Z drifts of p-MINFLUX setup (non-stabilized). For X and Y, the average displacement shown corresponds to the tracking of 7 AuNPs.

**
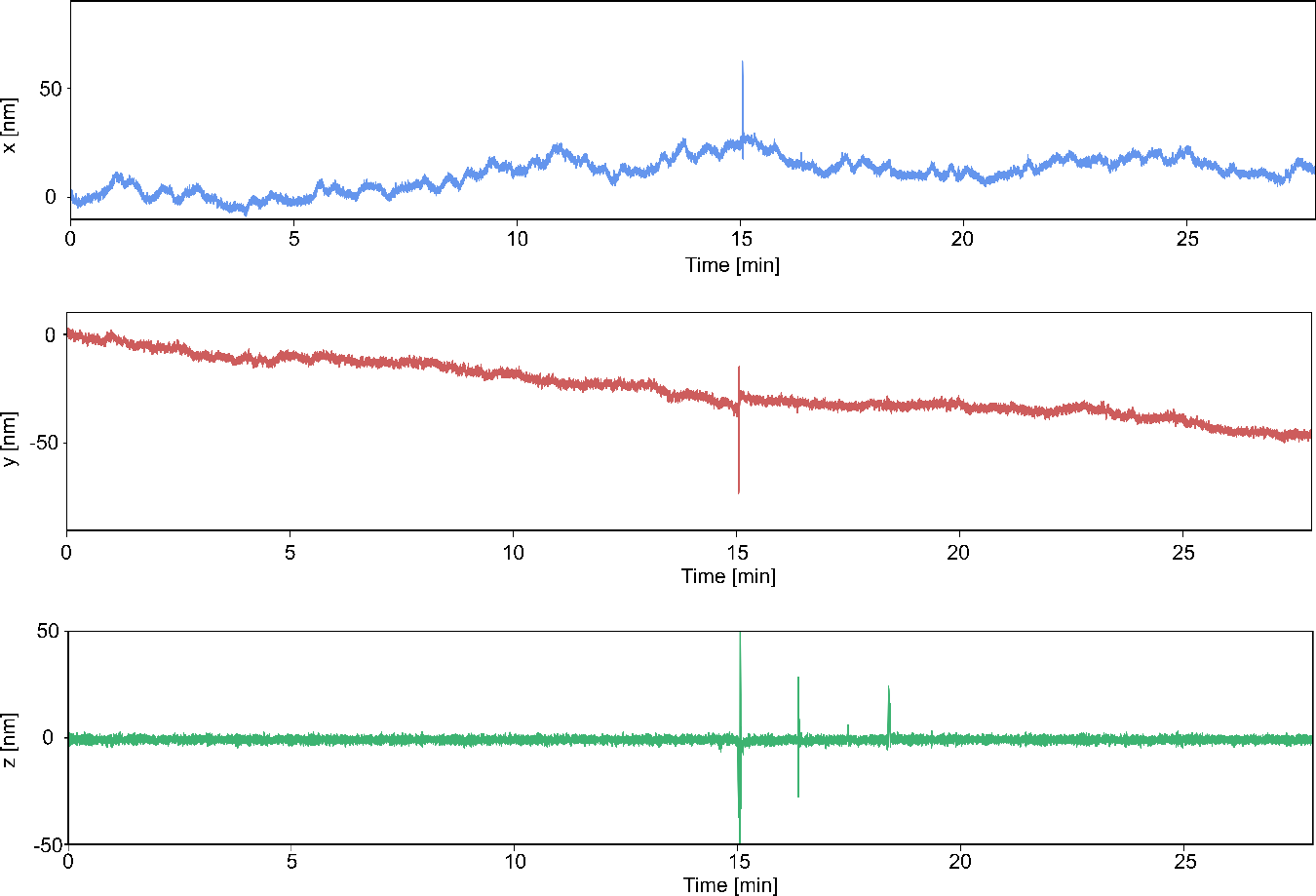
**

Figure S3. Sample X, Y and Z drifts of p-MINFLUX setup (Z stabilization only). For X and Y, the average displacement shown corresponds to the tracking of 6 AuNPs.


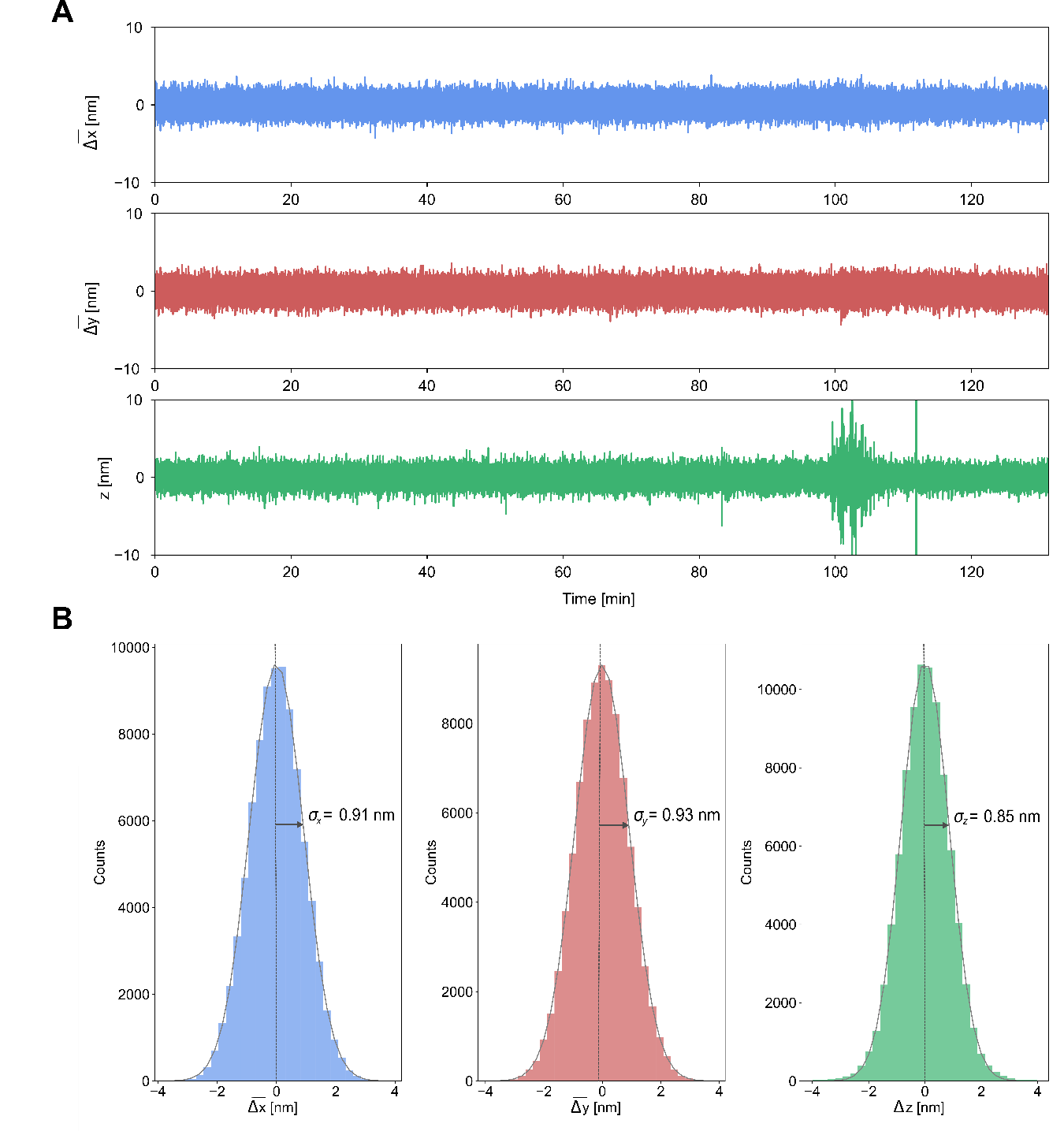


Figure S4. Performance of the stabilization system in the 3 dimensions for the p-MINFLUX setup. (A) Average displacements from the setpoint positions in X, Y and Z measured by the stabilization system for a period of 130 minutes using 7 AuNPs as fiducial markers. (B) Distribution of the displacements and gaussian fits (gray line) for each histogram. The parameter σ is the standard deviation of the gaussian fit and is indicated in each histogram.


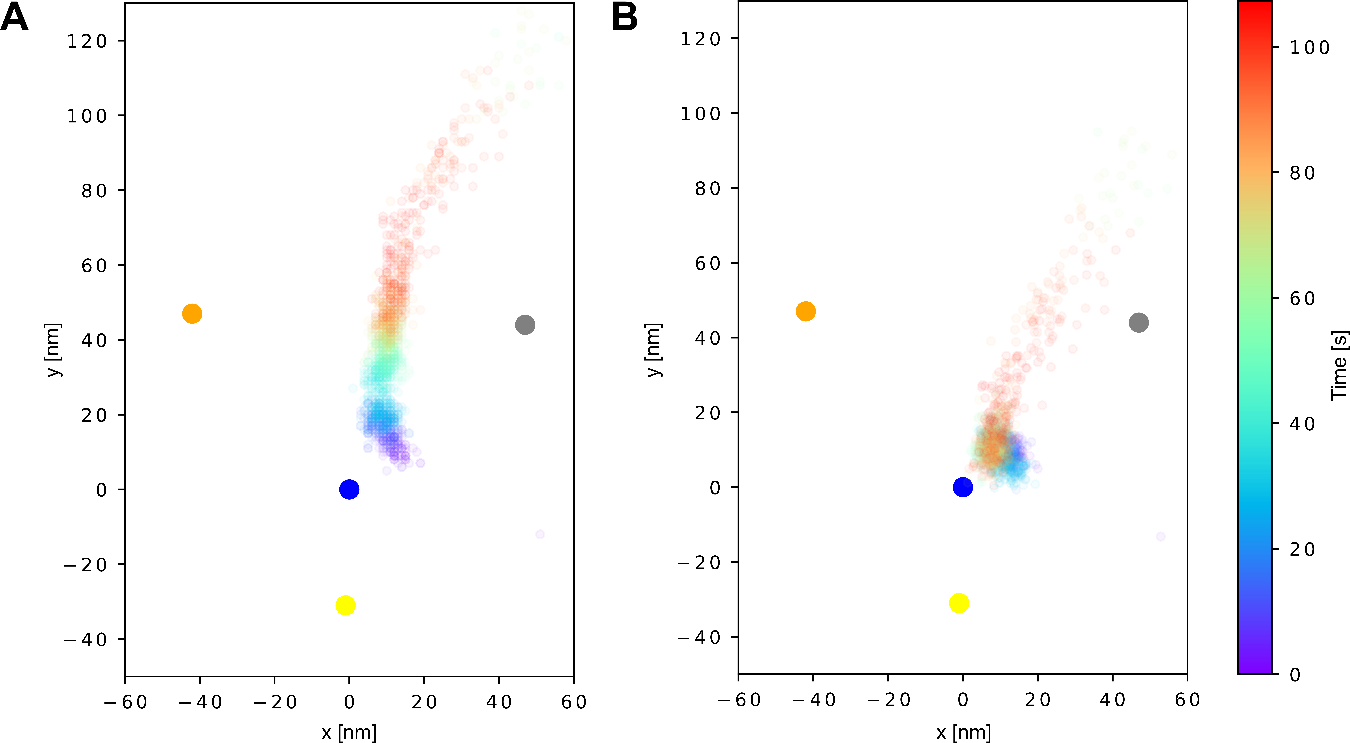


Figure S5. p-MINFLUX measurement with no active stabilization and post processing drift correction. (A) Localizations of a dynamic DNA origami structure with a single ATTO 643 fluorophore measured for 112 s (70 ms time bins, N = 1800). (B) Corrected localizations during post-processing using the measured XY drift data. The four solid dots indicate the excitation beam positions (EBPs) characteristic of the p-MINFLUX measurement scheme.

# Supplementary Tables

Table S1: DNA origami with a single ATTO 647N

| TAAAGACAAAGGCCGTAATAATTTTTTCAC |
| --- |
| TACCTGAGAACAAAATAACTATATAAAGAACG |
| GCACCAACATGACAACTCGGTTTATCAGCTTG |
| AGTACATAATTGCTTTAATAATGGCGAACGTT |
| GTACCGACAGCCAGTACGCAAGACGTAAATGC |
| CAGGCAAACGAAACGTTGAAGGGACCAGAGCA |
| GAATTGAGGAGGTGAGCAGAGATAATCCAGAA |
| AAACTATCGATTTAGTGCGCGTAACCACCA |
| ATCAAGAACAAAAGAATTCCTGATGAAGGAGC |
| CCAAGAAGACCGTGTATAAAGCGTGAATAA |
| GTGAGCCTCCTCACAGCGTGCCAGGCGGTATG |
| TTAAAAATAACAACTATTTACAAATGCACGTA |
| GCCGCCACTGGGAAGGTCTGCCAGAATTCGCG |
| CTGCCAGCTGAAATGTAAAGCAGCGCTTTC |
| AATTTTATCAGATAGCAATAGCAATATCACCG |
| AACATAGCATAAAGAAGAATATACGAGCCGTC |
| AATTTACCCTGTTTAGGAATCATCCTTGAA |
| GTTGGGCGGTTGTGTATCACGACGGAGGTGTC |
| CTCAGAAAGGCGGATCGTTCCAGAGGCAGG |
| TTCGAGCTTTAGTTTGTGGGGCGCATGCAATG |
| AGAATTAAAACAGGGAGAAGGCTTCAATAGCA |
| CTGTTTCCCGAGATATGAGAGAGTTGCAGC |
| AAATGAATGAGCGCTGCATGATAGTTTATT |
| TAAATCCGAACCGAACAGGACGGCGACAGA |
| TGATGCAGGGAACAAATTAAGTAAACAAAC |
| AAATACCCGGGTATTTATCAACTCCCAATC |
| GCCAGGGTGCAAGGCGACGGCGGACGTCGGAT |
| TGATAATCGTTCTAGCCGCAAGGAAGTAGTAG |
| GAATAGCCTGTGTGAAGTGAGCCATAAACA |
| TTTTGCGGCAGTCACACCACGCTGGGATTTTA |
| CACTCCAGCTCCGTGGACAGCGCCCGTCAGCG |
| GACGAGAACATAACGCAGACGACGATCAAAAA |
| TCCCGTATCATCATAGATGATGTGGGTAAC |
| CAGTACCCCGCCACCTAGTAAATGAATTTT |
| GGAGCACTACCGAACGAAGAATACAAGAACTC |
| CATAGGCTAGGGGGTAGTAGAAAGGAGTACCT |
| GCAAGGCATCGGCATGGAGGTTGTAAGCGT |
| CAATCAATTAAACACCGTATCATATTAATTAA |
| GTTGATATCTCAGGAGTAAACAACTTTCAACA |
| ATGAATCGTGGGCGCCAAAGGGCGAAAAACCG |
| ACCGATTGCGCCACCCAGCCACCAATTCTGAA |
| TCTGGTCCAACAGTGCGACCAGACAGGAAA |
| TTAATTGGTACGGTGATCATACTTGCGGGA |
| TTGTCACACATTGACATTTCGGTCGTTTTGCT |
| GTCTCGTCCAGCGCATGCTCGTTAACTCAC |
| CAATCGTCACGCGTGGCGGGGAAGAGCGGG |
| CAGCAAATCAAATATCAACCACCATATCAGAT |
| AAGCGGTCGCCTAATGAATTGTTACCTGCATC |
| TAGCAATGGAGCGGGGAAAGGA |
| TAGTCAGCATCAATTCCCAATTAATATGAT |
| CAAGATTCCGAGGAATAAGCCCATTAGAGC |
| AGATTCACAACAAAGAAAACCCTCATTTCAAT |
| AACTTAAAACCGTGCAGCGATCGGACCCCGGT |
| AGGGTAGCCAGCGAAAGAATAGAAAGGAACAA |
| TCTTCGCCAGTGCCACATTTGCGATGCTGA |
| CCTTGCTTACATCGGGAAATTATTCAATTCGA |
| TTAGGTTGTTCATCAAAATTATTCAATCAATA |
| TCAGAGCCGATTAGGATGATACAGCCAGAGCC |
| ACGACGAGCCAACATAATATATCCTCCGGC |
| CCTGAGTATTGTTAAATTTAAATTCCGGAAAC |
| AATAAGAAAATCGGCTAATAATATCCAGTTAC |
| CAGTACAAACTACAACGTGCCCGTAACCTATTCCGGAACC |
| ATTTCATCAACAAGCAAACATGTTAACGTCAA |
| ATTGTATAAGTCAAATTATTTTAAGAGCTGAA |
| AGACGATCGCTGGCAAGCAGCACACCGGAA |
| AGCCGGGAACCAGCTGTTAAACCGCCAGCA |
| CAGTTTGGTCGAATTCTCACTGCCGCTGGTAA |
| AAACTCCAAGTTGATTCTACTAATTAAAAATT |
| CAGCAAAACGTTTGCCAACCACCAGAGTGTAC |
| AACTAAACTCCTTTTACGAGAAAATGTTTA |
| CAAATAAGAATTGAGTACGCAATATATGGTTT |
| TGTTTTTAGCGCTTAAATCGGAACCCTAAAGG |
| GATGGCAAGGTTATATTAATTACAGGCAGAGG |
| GAGGACAGATGAACGGTGCGATTTTTGAGGAC |
| ACCAGCGGCCACCAGATCTTTTGACTCCTC |
| GACAGGAATGGTTGCTTTTTGGGGTCGAGG |
| AGCTAAACTTCCTCGTGCCCACTACGTGAACC |
| GACGACGAAGAGACGATAACCTACCGCAAG |
| TTTAGAAGTTTTGAATCCCTAAAAAACCGTTG |
| AAACTAGTAGCTATTAATACTTAGGCAAGG |
| CCTGGGGTCACGCTGGCCCTTATAAATCAAAA |
| TACCTTTTCTGTAAATAGATTAAGAGGCGTTA |
| GAGCCCCCGGCCTTGGAGGCCAAGCAGAAG |
| AACATAAACTGAACACTTAGCAAATATAAAAG |
| CGTGGACTCCAACGTCAGGGTGGTTTTTCTTT |
| CTTCACCGCGTTGCGCGTAATCATGCGCGCCT |
| TTTTCCCCGTCAGATATTGCGTTTTGAGGA |
| GATTAAGATTTTCATTACCATTAGGAGAAAGG |
| AAGAGAAGACCACCCTACGATCTAAAGTTTTG |
| TGCCGTAACCGCCAGCCCAGAATCTATTAACA |
| AATAGATACTGATAGGGCTATTTTGATTAG |
| ACAACATTTACCTTATGTACAGCTCCATGT |
| GGGAGTTTTTTTCATCGACCTGACCAGGCG |
| CCCACGCTCACTGTTTGCGGCCTCCCCGGG |
| TAATAACACGTGGCGACGCTAGGGCGCTGGCA |
| CAAGCCCAGTATTAAGACGGGGTCACCACCCT |
| GAAGCCTTTCCTGTAGTCATATGTTGCGGGCC |
| AACATTATGACCCTGTTTTGAGAGAAATATGC |
| TGGTGCTGACTGGTGTCGGTGCCCTCCGCTCA |
| GTTTCAGCGGAGTGAGACAGCATAGGTGTA |
| TTGCCGTTTGTGGTGCGCCCTGCGCGCTTTCC |
| ATAACCGAAAATACGTTATCATCGGAGTAATC |
| AATCGCGTGAATTACTTTTTAATTTAGTTA |
| AACGCTCAAATCAAGTTTTGACGAGCACGTAT |
| AACGTGCTAGGAGGCCCCTACATTACATTGGC |
| CCGCCTGAGTTGGCATGAGTAACTGATTGT |
| GACAATATTATTAGACATAGATTAAGTAACAG |
| TTACCGATCCAGAGCTTACCAAGTAGAAAC |
| TCCCTTACGTCTGGTCGCCTCCGGTAGCTCTC |
| AATCAAAATCCAATAATCTGGAAGTAATGCCG |
| CAGAGCACAGATATAAGCGCATGCTGAATA |
| CAGTCTCTGATTTTGCGTTTAGTACCGCCACC |
| ATCAAGTTGCACCGTATGGCAACACGTAGAAA |
| CCCACAAGAAACGATTTTTTGAAGTACCGCAC |
| GGTCTGAGTACTTCTGGAATACCAGTTGAAAG |
| AGTGTAGCGTCCATCACTGAGTAGGTGGCACA |
| GAGCCTTGAGATTTGAATGCCAGTAAATTG |
| GAGAGGGCATGTCAACCAGCTTAGATGGGC |
| TCACCAGAAACCTGTTTGAGGAAGAATGCG |
| CTCAGAACAGGGAGGGAGGTGAATTAGCTATC |
| ACAAATTCACAAGAAAGTCTTTCCCCAGCTAC |
| GACTGGATAGCGTCCAAAAACGAAGTCATTTT |
| TTTAGAAGAACGCCACCCAAAACAGGCTGC |
| CCGGAGACAGCAAATATCAGCTCAGAAGATCG |
| GCCGCCAGATCAATAGAGCACCATAGAGATAA |
| TCTATCAGGGCGATGTAGAATCGAGGCGGT |
| TGAGTTTTATTTCGGATAAACACCGCCACC |
| TAACAACCAATAAACAGCCGTTGCGAACCT |
| GTGCACTCCCGGCAAACCGTCGGTGAGGTGGA |
| GCCTCCCTTCATTAAAAGGTAATTTTAAGA |
| CATTAACAATCAGGTCGGATTAGAATTCATCA |
| ACGGAAAACAGTATCCGCCATTACAGGAAG |
| TGGTAATTTAGCGTACATTTTCAGGGATAG |
| CTTATTAGTCACCAGTAAAATTCAATAACGGA |
| TTGTGAATATTACAGATAGTAATGACCATA |
| GCATCGTATTTCTGCTAGCTTTCAGGTGCCAT |
| ATACCCAAGCGGGAGGTTTTGTTTCAGCTAAT |
| AAGGAAAAGTTGCTATTATTTAAATAGATA |
| AAACAGAAGATAGCTTCGTCGCTATGCGTTAT |
| TCACCGACGGAACCAGTCAGAGCCAGTGCCTT |
| GATAGTTGCGCCGACACTAAAACGCAAGCGCGACCCAAAT |
| AAAAATCTATACCAGTCTGACCAACGGTCAAT |
| AAAATAAAAACAATGACGAACAAAGACATTCA |
| CTTATTACACGCGAGGCCTTTACACTGTCCAG |
| TTAAACAGTTCAGAAAGATAAGAGCTAACGGA |
| ATTAAATGTGAGCGAGATGAACGGGAAAGGGG |
| TTGGATTAAGACTACCCTTTTTTAGCCATATT |
| GTTGAGATATGGTTTATTCATCAACCTGATAA |
| GGGTAATAATAGCAGCGTTTTATTTATTTT |
| CAACGTAACGTTTACCCAAAAGGACGTTTTAA |
| ATAAATACATAAAGGATCAGTATTGGGAAG |
| CCAATAGCCCTCATACACCATCCTGCGAAC |
| CAATATTAAGCACTAATGCGCCGCTACAGGGC |
| TCATCGAGTTCTGACCTGAGAATCATGGAAAC |
| TACCGAGCAACAAGAGGCAACAGCTGATTGCC |
| CATCGTAGCTTTTTCAGTAATTTATTTAACAA |
| AAGAGTCCCATATCAAGAAACATATCTTTA |
| ATAAAACAGAAGGTTACCTTTGCCAAGGGTTA |
| ACCCTCAGAACGGCTAGGCGCAGACTTTGAAA |
| TACTTAGCCGGAACGACAGAGGCTTAAGAACT |
| CTCATAGAAGTTTTAAGGCTGACATAATCA |
| AGCAAATTAAAGCTAGCCTGAGAATATAAA |
| CTGACCTTATTAAATTCTAAAAATAACGGA |
| CTGTATGGGAATTTACAAGTGCCGCTGTAGCG |
| TACAACGTAATTGTAAACCATCGCCCACGC |
| GCGTACTACGGTACGCATTGCATAATAAAA |
| ATCACCCATGGAAATAGATTAAAGAGAGCCAG |
| AGTCCTGATTACCAGTGATAAATAACGCTGAG |
| TTCTAAGAGCAGTATGCCTGAACAGAAACCAT |
| TCACCGTAAAGTATAGGCCAGAATCAAACAAA |
| ATTCAACCAGAAAAGCTCAAAAATTTTGAGGG |
| CAGAGCCCCAAAGACTTTGGGAAATAATAA |
| CAATTCCAGGCAAAATTTTGCCCC |
| AACAGTAGCGCCTGTTAAACCAAGCCTTAAAT |
| CAGCATCATCCGCCGGGTCATACCTCGCGTCC |
| TTTGCCAGGGCTGACCATTTCAACTTCCATTA |
| GTTGAAAAGAAATCCGGAGGAAGTTTTAATCA |
| CCGGCGAATCACTTGCCGCAAATTCATCGCCA |
| TTGCGTATGCCAACGCCCTGTTCTGGGGGTTT |
| TTCGCCTGAATCAATAATTTATCAATGGTTTG |
| TCAGACGAAGACACCATCACCAATAAGTCAGA |
| CGACGGCTATTACGCTTGGTGTTCATCAAC |
| GCGGGCCGTTTTCACGGCGCGGTTCTGCATTA |
| CATTTTCGAAAAGGTAACCGCGCCATCCGGTA |
| GAGTAACAGCCTGTAGCCATGTACCGTAACAC |
| CAAAGAATTAGCAAAACATGTTTTATCTACAA |
| AAATCACCTTGAGCCAAAAAGGGCGTTACCAG |
| ACAATCGGGCGCCATTGGCCTCAGTTTTTTAA |
| CGAGAAAAGAATCATTAAGTAATTGAGAGAAT |
| TTTCATTCAGAGGCGTATAATCCATTATCA |
| CTAAAGGAATTGCGAACTTTTGCGGGATCGTC |
| AAGGTGGAAGCAAAGAACCAGATCAACTAA |
| GGAATTAAAAAAAGCATTGCAGTCACCTTG |
| CATACATGCCAGACGTCTCAGAACCGCCACCC |
| CGTTTTCCGGAAACGCGGAATATAAGACTC |
| GAGCAAGACAGCCATATTTTGCACTTATCATT |
| TGCGGATGTAGCTCAATTAAGCAAGTACCAAA |
| CACCCGCCTAATCAGTCTGGTAATGAACCCTT |
| CTTTCGAGGATTATACAAAGAGGCCTTGCCCT |
| GAACCTAAATAGTGATATGTGACAACGCTC |
| AATGGGATAGGTCACGCAGCTGGCTAATCGTA |
| AGGCTATCGAGAATCGTAACAACCTTGACCGT |
| ATACATATTCATTGACTTAATTTAGACGGG |
| TTGACAAGCGAGAGGACCACATCCGGAAGC |
| TCTGGCCTTATTTCAATGATAAATTTTCATTC |
| CGATAGCATGCCTTTAGATATTCAGGAAAGCG |
| TGCAGATAACACCAGATATTCATTAAACAAAG |
| TCTCCGTAATCCAATATAAGAGAGTCTGGA |
| GAGTAGATTCAAAGCGCGGATTGCATAAAAAC |
| CTGAACCTGAAAAATCGATTATTTTTGACGCT |
| ATTAATTTCTGGCCAAGCGGTCAGCTGAGAAG |
| AGTCGGGTGAGACGGTCCACTATTAAAGAA |
| AAACGCAATTGGCCTTGCGTCAGATCGAGAGG |
| ATTGTGTCTCTCCAAAGTCGCTGAGGCTTGCA |
| AGTGATGAAGGGTAAATACGGCTGTTGTAAAA |
| AATGCCAAGGTTTCTTGTGTCACTGGTCATAG |
| CATAAGGTCATTAAACCCGGAATCGGAACG |
| CATCCTCAAGCGGTGCGTTCAGCAGTGTAAAG |
| AAAGTAAGCCTGAATCCTAATTTGCCCATCCT |
| GGGACATTTAAAAGTTAATCAACAAGTTACAA |
| CCCGACTTAAGAACTGAATATCAGTACCATTA |
| TCGTCTTTGCTTTTGATTAGCGGGATAGCCCC |
| MODIFIED |
| CAACTCGGAAAGCGTAACCACCCCGAGTAATTT**[ATTO542]** |
| CAAAATAGAACCGGAACGAGTACTACGAAGTTT**[ATTO647N]** |
| **[biotin]**TTTAAGAGTCTGGTCACGCAGCTTGACGGGGAAAG |
| **[biotin]**TTTACATGAAAATAGGAACCATTCCACAGACAGCC |
| **[biotin]**TTTGCAGAACGGGCTTAATTAAATTTAAAATCATA |
| **[biotin]**TTTGCAAACAAAGGTCATTAATCGGTTTAAAGCCT |
| **[biotin]**TTTATTAATTGCCTGGCCCGGGTTGAGTGTTGTTC |
| **[biotin]**TTTAACGGGTATATATTCGAAAAAGGCTCCAAAAG |

Table S2: Dynamic DNA origami with a single ATTO 643

| TAAAGACAAAGGCCGTAATAATTTTTTCAC |
| --- |
| TACCTGAGAACAAAATAACTATATAAAGAACG |
| GCACCAACATGACAACTCGGTTTATCAGCTTG |
| AGTACATAATTGCTTTAATAATGGCGAACGTT |
| GTACCGACAGCCAGTACGCAAGACGTAAATGC |
| CAGGCAAACGAAACGTTGAAGGGACCAGAGCA |
| GAATTGAGGAGGTGAGCAGAGATAATCCAGAA |
| AAACTATCGATTTAGTGCGCGTAACCACCA |
| ATCAAGAACAAAAGAATTCCTGATGAAGGAGC |
| CCAAGAAGACCGTGTATAAAGCGTGAATAA |
| GTGAGCCTCCTCACAGCGTGCCAGGCGGTATG |
| TTAAAAATAACAACTATTTACAAATGCACGTA |
| GCACTCAAGCGGGGTCCGCACAGGTAAAAAAA |
| CTGCCAGCTGAAATGTAAAGCAGCGCTTTC |
| TGGGTAAACGGCAGCACGCGGTCCGCGGATCA |
| AATTTTATCAGATAGCAATAGCAATATCACCG |
| AACATAGCATAAAGAAGAATATACGAGCCGTC |
| AATTTACCCTGTTTAGGAATCATCCTTGAA |
| GTTGGGCGGTTGTGTATCACGACGGAGGTGTC |
| CTCAGAAAGGCGGATCGTTCCAGAGGCAGG |
| TTCGAGCTTTAGTTTGTGGGGCGCATGCAATG |
| AGAATTAAAACAGGGAGAAGGCTTCAATAGCA |
| GAGAGGGCATGTCAACCAGCTTAGATGGGC |
| CTCAGAACAGGGAGGGAGGTGAATTAGCTATC |
| CTGTTTCCCGAGATATGAGAGAGTTGCAGC |
| AAATGAATGAGCGCTGCATGATAGTTTATT |
| TAAATCCGAACCGAACAGGACGGCGACAGA |
| TGATGCAGGGAACAAATTAAGTAAACAAAC |
| AAATACCCGGGTATTTATCAACTCCCAATC |
| GCCAGGGTGCAAGGCGACGGCGGACGTCGGAT |
| CAACTCGGAAAGCGTAACCACCCCGAGTAA |
| GAATAGCCTGTGTGAAGTGAGCCATAAACA |
| TTTTGCGGCAGTCACACCACGCTGGGATTTTA |
| CACTCCAGCTCCGTGGACAGCGCCCGTCAGCG |
| GACGAGAACATAACGCAGACGACGATCAAAAA |
| TCCCGTATCATCATAGATGATGTGGGTAAC |
| CAGTACCCCGCCACCTAGTAAATGAATTTT |
| GGAGCACTACCGAACGAAGAATACAAGAACTC |
| CATAGGCTAGGGGGTAGTAGAAAGGAGTACCT |
| GCAAGGCATCGGCATGGAGGTTGTAAGCGT |
| CAATCAATTAAACACCGTATCATATTAATTAA |
| GTTGATATCTCAGGAGTAAACAACTTTCAACA |
| ATGAATCGTGGGCGCCAAAGGGCGAAAAACCG |
| ACCGATTGCGCCACCCAGCCACCAATTCTGAA |
| TCTGGTCCAACAGTGCGACCAGACAGGAAA |
| TTGTCACACATTGACATTTCGGTCGTTTTGCT |
| GTCTCGTCCAGCGCATGCTCGTTAACTCAC |
| CAATCGTCACGCGTGGCGGGGAAGAGCGGG |
| CAGCAAATCAAATATCAACCACCATATCAGAT |
| AAGCGGTCGCCTAATGAATTGTTACCTGCATC |
| TAGCAATGGAGCGGGGAAAGGA |
| TAGTCAGCATCAATTCCCAATTAATATGAT |
| CAAGATTCCGAGGAATAAGCCCATTAGAGC |
| AGATTCACAACAAAGAAAACCCTCATTTCAAT |
| AACTTAAAACCGTGCAGCGATCGGACCCCGGT |
| AGGGTAGCCAGCGAAAGAATAGAAAGGAACAA |
| TCTTCGCCAGTGCCACATTTGCGATGCTGA |
| CCTTGCTTACATCGGGAAATTATTCAATTCGA |
| TTAGGTTGTTCATCAAAATTATTCAATCAATA |
| TCAGAGCCGATTAGGATGATACAGCCAGAGCC |
| ACGACGAGCCAACATAATATATCCTCCGGC |
| GCAACTGTGGGAACGGCAGAAACAGTTTTTTC |
| CCTGAGTATTGTTAAATTTAAATTCCGGAAAC |
| AATAAGAAAATCGGCTAATAATATCCAGTTAC |
| CAGTACAAACTACAACGTGCCCGTAACCTATTCCGGAACC |
| ATTTCATCAACAAGCAAACATGTTAACGTCAA |
| ATTGTATAAGTCAAATTATTTTAAGAGCTGAA |
| AGACGATCGCTGGCAAGCAGCACACCGGAA |
| AGCCGGGAACCAGCTGTTAAACCGCCAGCA |
| CAGTTTGGTCGAATTCTCACTGCCGCTGGTAA |
| CAGCAAAACGTTTGCCAACCACCAGAGTGTAC |
| AGGCTATCGAGAATCGTAACAACCTTGACCGT |
| AACTAAACTCCTTTTACGAGAAAATGTTTA |
| CAAATAAGAATTGAGTACGCAATATATGGTTT |
| TGTTTTTAGCGCTTAAATCGGAACCCTAAAGG |
| GATGGCAAGGTTATATTAATTACAGGCAGAGG |
| GAGGACAGATGAACGGTGCGATTTTTGAGGAC |
| ACCAGCGGCCACCAGATCTTTTGACTCCTC |
| GACAGGAATGGTTGCTTTTTGGGGTCGAGG |
| AGCTAAACTTCCTCGTGCCCACTACGTGAACC |
| GACGACGAAGAGACGATAACCTACCGCAAG |
| TTTAGAAGTTTTGAATCCCTAAAAAACCGTTG |
| CCTGGGGTCACGCTGGCCCTTATAAATCAAAA |
| TACCTTTTCTGTAAATAGATTAAGAGGCGTTA |
| GAGCCCCCGGCCTTGGAGGCCAAGCAGAAG |
| AACATAAACTGAACACTTAGCAAATATAAAAG |
| CGTGGACTCCAACGTCAGGGTGGTTTTTCTTT |
| CTTCACCGCGTTGCGCGTAATCATGCGCGCCT |
| TTTTCCCCGTCAGATATTGCGTTTTGAGGA |
| GATTAAGATTTTCATTACCATTAGGAGAAAGG |
| AAGAGAAGACCACCCTACGATCTAAAGTTTTG |
| TGCCGTAACCGCCAGCCCAGAATCTATTAACA |
| AATAGATACTGATAGGGCTATTTTGATTAG |
| ACAACATTTACCTTATGTACAGCTCCATGT |
| GGGAGTTTTTTTCATCGACCTGACCAGGCG |
| CCCACGCTCACTGTTTGCGGCCTCCCCGGG |
| CAAAATAGAACCGGAACGAGTACTACGAAG |
| TAATAACACGTGGCGACGCTAGGGCGCTGGCA |
| CAAGCCCAGTATTAAGACGGGGTCACCACCCT |
| GAAGCCTTTCCTGTAGTCATATGTTGCGGGCC |
| AACATTATGACCCTGTTTTGAGAGAAATATGC |
| TGGTGCTGACTGGTGTCGGTGCCCTCCGCTCA |
| GTTTCAGCGGAGTGAGACAGCATAGGTGTA |
| TTGCCGTTTGTGGTGCGCCCTGCGCGCTTTCC |
| ATAACCGAAAATACGTTATCATCGGAGTAATC |
| AATCGCGTGAATTACTTTTTAATTTAGTTA |
| AACGCTCAAATCAAGTTTTGACGAGCACGTAT |
| AACGTGCTAGGAGGCCCCTACATTACATTGGC |
| CCGCCTGAGTTGGCATGAGTAACTGATTGT |
| GACAATATTATTAGACATAGATTAAGTAACAG |
| TTACCGATCCAGAGCTTACCAAGTAGAAAC |
| TCCCTTACGTCTGGTCGCCTCCGGTAGCTCTC |
| AATCAAAATCCAATAATCTGGAAGTAATGCCG |
| CAGAGCACAGATATAAGCGCATGCTGAATA |
| CAGTCTCTGATTTTGCGTTTAGTACCGCCACC |
| ATCAAGTTGCACCGTATGGCAACACGTAGAAA |
| CCCACAAGAAACGATTTTTTGAAGTACCGCAC |
| GGTCTGAGTACTTCTGGAATACCAGTTGAAAG |
| AGTGTAGCGTCCATCACTGAGTAGGTGGCACA |
| GAGCCTTGAGATTTGAATGCCAGTAAATTG |
| TCACCAGAAACCTGTTTGAGGAAGAATGCG |
| ACAAATTCACAAGAAAGTCTTTCCCCAGCTAC |
| GACTGGATAGCGTCCAAAAACGAAGTCATTTT |
| TTTAGAAGAACGCCACCCAAAACAGGCTGC |
| CCGGAGACAGCAAATATCAGCTCAGAAGATCG |
| GCCGCCAGATCAATAGAGCACCATAGAGATAA |
| TCTATCAGGGCGATGTAGAATCGAGGCGGT |
| TGAGTTTTATTTCGGATAAACACCGCCACC |
| TAACAACCAATAAACAGCCGTTGCGAACCT |
| GTGCACTCCCGGCAAACCGTCGGTGAGGTGGA |
| GCCTCCCTTCATTAAAAGGTAATTTTAAGA |
| CATTAACAATCAGGTCGGATTAGAATTCATCA |
| ACGGAAAACAGTATCCGCCATTACAGGAAG |
| TGGTAATTTAGCGTACATTTTCAGGGATAG |
| CTTATTAGTCACCAGTAAAATTCAATAACGGA |
| TTGTGAATATTACAGATAGTAATGACCATA |
| GCATCGTATTTCTGCTAGCTTTCAGGTGCCAT |
| ATACCCAAGCGGGAGGTTTTGTTTCAGCTAAT |
| AAGGAAAAGTTGCTATTATTTAAATAGATA |
| AAACAGAAGATAGCTTCGTCGCTATGCGTTAT |
| TCACCGACGGAACCAGTCAGAGCCAGTGCCTT |
| GATAGTTGCGCCGACACTAAAACGCAAGCGCGACCCAAAT |
| AAAAATCTATACCAGTCTGACCAACGGTCAAT |
| AAAATAAAAACAATGACGAACAAAGACATTCA |
| CTTATTACACGCGAGGCCTTTACACTGTCCAG |
| TAATGCTGGCTTAGAGATCCCCCTGAATCGTC |
| TTAAACAGTTCAGAAAGATAAGAGCTAACGGA |
| ATTAAATGTGAGCGAGATGAACGGGAAAGGGG |
| AATGCCAAGGTTTCTTGTGTCACTGGTCATAG |
| TTGGATTAAGACTACCCTTTTTTAGCCATATT |
| GTTGAGATATGGTTTATTCATCAACCTGATAA |
| GGGTAATAATAGCAGCGTTTTATTTATTTT |
| CAACGTAACGTTTACCCAAAAGGACGTTTTAA |
| ATAAATACATAAAGGATCAGTATTGGGAAG |
| CCAATAGCCCTCATACACCATCCTGCGAAC |
| CAATATTAAGCACTAATGCGCCGCTACAGGGC |
| TCATCGAGTTCTGACCTGAGAATCATGGAAAC |
| TACCGAGCAACAAGAGGCAACAGCTGATTGCC |
| CATCGTAGCTTTTTCAGTAATTTATTTAACAA |
| AAGAGTCCCATATCAAGAAACATATCTTTA |
| ATAAAACAGAAGGTTACCTTTGCCAAGGGTTA |
| ACCCTCAGAACGGCTAGGCGCAGACTTTGAAA |
| TACTTAGCCGGAACGACAGAGGCTTAAGAACT |
| CTCATAGAAGTTTTAAGGCTGACATAATCA |
| AGCAAATTAAAGCTAGCCTGAGAATATAAA |
| CTGACCTTATTAAATTCTAAAAATAACGGA |
| CTGTATGGGAATTTACAAGTGCCGCTGTAGCG |
| TACAACGTAATTGTAAACCATCGCCCACGC |
| GCGTACTACGGTACGCATTGCATAATAAAA |
| ATCACCCATGGAAATAGATTAAAGAGAGCCAG |
| AGTCCTGATTACCAGTGATAAATAACGCTGAG |
| GCCGCCACTGGGAAGGTCTGCCAGAATTCGCG |
| TTCTAAGAGCAGTATGCCTGAACAGAAACCAT |
| GATGTGCTTTTCCCAGCATCGACACGGCCTTT |
| TCACCGTAAAGTATAGGCCAGAATCAAACAAA |
| ATTCAACCAGAAAAGCTCAAAAATTTTGAGGG |
| CAGAGCCCCAAAGACTTTGGGAAATAATAA |
| CAATTCCAGGCAAAATTTTGCCCC |
| AACAGTAGCGCCTGTTAAACCAAGCCTTAAAT |
| CAGCATCATCCGCCGGGTCATACCTCGCGTCC |
| TTTGCCAGGGCTGACCATTTCAACTTCCATTA |
| GTTGAAAAGAAATCCGGAGGAAGTTTTAATCA |
| CCGGCGAATCACTTGCCGCAAATTCATCGCCA |
| TTGCGTATGCCAACGCCCTGTTCTGGGGGTTT |
| TTCGCCTGAATCAATAATTTATCAATGGTTTG |
| TCAGACGAAGACACCATCACCAATAAGTCAGA |
| CGACGGCTATTACGCTTGGTGTTCATCAAC |
| GCGGGCCGTTTTCACGGCGCGGTTCTGCATTA |
| CATTTTCGAAAAGGTAACCGCGCCATCCGGTA |
| GAGTAACAGCCTGTAGCCATGTACCGTAACAC |
| CAAAGAATTAGCAAAACATGTTTTATCTACAA |
| AAATCACCTTGAGCCAAAAAGGGCGTTACCAG |
| ACAATCGGGCGCCATTGGCCTCAGTTTTTTAA |
| CGAGAAAAGAATCATTAAGTAATTGAGAGAAT |
| TTTCATTCAGAGGCGTATAATCCATTATCA |
| CTAAAGGAATTGCGAACTTTTGCGGGATCGTC |
| AAGGTGGAAGCAAAGAACCAGATCAACTAA |
| GGAATTAAAAAAAGCATTGCAGTCACCTTG |
| CATACATGCCAGACGTCTCAGAACCGCCACCC |
| CGTTTTCCGGAAACGCGGAATATAAGACTC |
| GAGCAAGACAGCCATATTTTGCACTTATCATT |
| TGCGGATGTAGCTCAATTAAGCAAGTACCAAA |
| CACCCGCCTAATCAGTCTGGTAATGAACCCTT |
| CTTTCGAGGATTATACAAAGAGGCCTTGCCCT |
| GAACCTAAATAGTGATATGTGACAACGCTC |
| AATGGGATAGGTCACGCAGCTGGCTAATCGTA |
| ATACATATTCATTGACTTAATTTAGACGGG |
| TTGACAAGCGAGAGGACCACATCCGGAAGC |
| TCTGGCCTTATTTCAATGATAAATTTTCATTC |
| CGATAGCATGCCTTTAGATATTCAGGAAAGCG |
| TGCAGATAACACCAGATATTCATTAAACAAAG |
| TCTCCGTAATCCAATATAAGAGAGTCTGGA |
| GAGTAGATTCAAAGCGCGGATTGCATAAAAAC |
| CTGAACCTGAAAAATCGATTATTTTTGACGCT |
| ATTAATTTCTGGCCAAGCGGTCAGCTGAGAAG |
| AGTCGGGTGAGACGGTCCACTATTAAAGAA |
| AAACGCAATTGGCCTTGCGTCAGATCGAGAGG |
| ATTGTGTCTCTCCAAAGTCGCTGAGGCTTGCA |
| AGTGATGAAGGGTAAATACGGCTGTTGTAAAA |
| CATAAGGTCATTAAACCCGGAATCGGAACG |
| GGCTCATTACGTTAATATACTGCGCAAATGCT |
| CATCCTCAAGCGGTGCGTTCAGCAGTGTAAAG |
| AAAGTAAGCCTGAATCCTAATTTGCCCATCCT |
| GGGACATTTAAAAGTTAATCAACAAGTTACAA |
| CCCGACTTAAGAACTGAATATCAGTACCATTA |
| TCGTCTTTGCTTTTGATTAGCGGGATAGCCCC |
| MODIFIED |
| AAACTCCAAGTTGATTCTACTAATAGTAGTAGTTTTTTTTTCGGGCATTTA **[At643]** |
| GGCTTGAGTTAGGAATCTTTTGCAAAAGAAGTTTTTAAATGC |
| AAACTAGTAGCTATTAATACTTTTGCGGGATTTTAAATGC |
| CATATAACACAGGTCATTTACCCTGACTATTA |
| TGATAATCGTTCTAGCCGCAAGGATAAAAATT |
| TTAATTGGTACGGTGATCATACAGGCAAGG |
| **[biotin]**TTTAAGAGTCTGGTCACGCAGCTTGACGGGGAAAG |
| **[biotin]**TTTACATGAAAATAGGAACCATTCCACAGACAGCC |
| **[biotin]**TTTGCAGAACGGGCTTAATTAAATTTAAAATCATA |
| **[biotin]**TTTGCAAACAAAGGTCATTAATCGGTTTAAAGCCT |
| **[biotin]**TTTATTAATTGCCTGGCCCGGGTTGAGTGTTGTTC |
| **[biotin]**TTTAACGGGTATATATTCGAAAAAGGCTCCAAAAG |
